# Novel Au(I)- and Ag(I)-NHC Complexes with *N*-Boc-Protected Proline as Potential Candidates for Neurodegenerative Disorders

**DOI:** 10.3390/ijms26136116

**Published:** 2025-06-25

**Authors:** Jessica Ceramella, Assunta D’Amato, Francesca Procopio, Annaluisa Mariconda, Daniel Chavarria, Domenico Iacopetta, Francesco Ortuso, Pasquale Longo, Fernanda Borges, Maria Stefania Sinicropi

**Affiliations:** 1 Department of Pharmacy, Health and Nutritional Sciences, University of Calabria, Via Pietro Bucci, 87036 Arcavacata di Rende, Italy; jessica.ceramella@unical.it (J.C.); s.sinicropi@unical.it (M.S.S.); 2 Department of Chemistry and Biology “A. Zambelli”, University of Salerno, Via Giovanni Paolo II 132, 84084 Fisciano, Italy; asdamato@unisa.it (A.D.); plongo@unisa.it (P.L.); 3 Dipartimento di Scienze della Salute, Università “Magna Græcia” di Catanzaro, Viale Europa, 88100 Catanzaro, Italy; francesca.procopio001@studenti.unicz.it (F.P.); ortuso@unicz.it (F.O.); 4 Department of Basic and Applied Sciences (DISBA), University of Basilicata, Via Dell’Ateneo Lucano 10, 85100 Potenza, Italy; annaluisa.mariconda@unibas.it; 5 CIQUP-IMS—Centro de Investigação em Química da Universidade do Porto, Institute of Molecular Sciences, Department of Chemistry and Biochemistry, Faculty of Sciences, University of Porto, Rua do Campo Alegre s/n, 4169-007 Porto, Portugal; 6 MedInUP, Center for Drug Discovery and Innovative Medicines, University of Porto, 4200-319 Porto, Portugal; fborges@fc.up.pt; 7 Department of Chemistry and Biochemistry, Faculty of Sciences, University of Porto, Rua do Campo Alegre s/n, 4169-007 Porto, Portugal; 8 Department of Biomedicine-Pharmacology and Therapeutics Unit, Faculty of Medicine, University of Porto, 4200-319 Porto, Portugal

**Keywords:** NHC complexes, silver(I)/gold(I), proline, neurodegenerative diseases, MAO inhibitors, cholinesterase inhibitors, oxidative stress, anti-inflammation properties

## Abstract

Neurodegenerative diseases (NDDs), including Alzheimer’s disease (AD) and Parkinson’s disease (PD), are characterized by progressive neuronal dysfunction and loss and represent a significant global health challenge. Oxidative stress, neuroinflammation, and neurotransmitter dysregulation, particularly affecting acetylcholine (ACh) and monoamines, are key hallmarks of these conditions. The current therapeutic strategies targeting cholinergic and monoaminergic systems have some limitations, highlighting the need for novel approaches. Metallodrugs, especially ruthenium and platinum complexes, are gaining attention for their therapeutic use. Among metal complexes, gold(I) and silver(I) *N*-heterocyclic carbene (NHC) complexes exhibit several biological activities, but their application in NDDs, particularly as monoamine oxidase (MAO) inhibitors, remains largely unexplored. To advance the understanding of this field, we designed, synthesized, and evaluated the biological activity of a new series of Au(I) and Ag(I) complexes stabilized by NHC ligands and bearing a carboxylate salt of tert-butyloxycarbonyl (Boc)-*N*-protected proline as an anionic ligand. Through in silico and in vitro studies, we assessed their potential as acetylcholinesterase (AChE) and MAO inhibitors, as well as their antioxidant and anti-inflammatory properties, aiming to contribute to the development of potential novel therapeutic agents for NDD management.

## 1. Introduction

Neurodegenerative diseases (NDDs), given their substantial impact on the ageing population, are among the leading causes of disability and morbidity worldwide. These diseases are defined by a continuous decline in neuronal function, culminating in brain atrophy and significant functional limitations [1].

The most commonly known NDDs, Alzheimer’s and Parkinson’s diseases (AD and PD, respectively), are progressive and multifactorial forms of neurodegeneration. These diseases share several key characteristics, involving a complex interplay of genetic, environmental, and lifestyle factors, which lead to a progressive decline in physical, cognitive, and emotional functions. The underlying mechanisms include the degeneration of brain cells, accompanied with chronic neuroinflammation, neuronal death, and a gradual loss of brain tissue [1].

The “switch on” of the inflammatory cascade triggers multiple pathways and responses, like the production of reactive oxygen species (ROS) and cytokines, resulting in oxidative stress. Excessive ROS generation damages cellular components like proteins, lipids, and DNA, leading to neuronal dysfunction and death and contributing to the progressive decline observed in both AD and PD [2].

Together with oxidative stress and neuroinflammation, the deregulation of neurotransmitter systems represents an important hallmark that significantly contributes to the observed cognitive and motor deficits [3].

Particularly, the homeostasis of neurotransmitters including acetylcholine (ACh), butyrylcholine (BCh), and monoamines (such as dopamine, serotonin, and norepinephrine) plays a crucial role in various neurological and psychiatric conditions.

In AD, the loss of cholinergic neurons leads to reduced acetylcholine (ACh) levels, contributing to cognitive decline. Similarly, imbalances in monoamine neurotransmitters are linked to a range of disorders (e.g., PD, depression, anxiety, panic disorder, and attention-deficit/hyperactivity disorders).

The current therapeutic strategies, such as the inhibition of acetylcholinesterase (AChE), aim to increase the ACh availability to prevent cognitive decline and memory loss [4]. AChE is the main enzyme that hydrolyzes ACh; however, the effectiveness of acetylcholinesterase inhibitors (AChEIs) in treating ongoing AD may be limited by significant neuronal damage and the substantial, often irreversible, loss of AChE activity (>90%). Conversely, butyrylcholinesterase (BChE) levels can increase to up to 120% above physiological levels in ongoing AD. This BChE upregulation suggests the potential compensatory role of BChE inhibitors in disease progression [5].

Moreover, the development of monoamine oxidase inhibitors (MAOIs) in medicinal chemistry constitutes a critical area of investigation, driven by the need for safer and more effective treatments for various neurological and psychiatric disorders.

MAOIs are a class of drugs that inhibit the enzymes monoamine oxidase A (MAO-A) and/or B (MAO-B). MAO-A primarily metabolizes serotonin and norepinephrine; thus, its inhibition is therapeutically exploited to treat depression, anxiety, and social phobia. On the other hand, MAO-B inhibitors are employed in PD since this enzyme predominantly metabolizes dopamine [6].

In addition, elevated MAO expression and activity have been associated with increased oxidative stress, since the metabolism of monoamines generates their corresponding aldehydes, with the concomitant formation of H_2_O_2_ and ammonia as byproducts. In particular, H_2_O_2_ induces mitochondrial damage and neuronal apoptosis [7].

Recently, metallodrugs have garnered considerable attention within the scientific community as a potential therapeutic approach for treating NDDs [8,9]. Among the diverse complexes, ruthenium- and platinum-based metal complexes have emerged as prominent candidates for NDD therapy. The current research primarily focuses on AD, with an emphasis on Aβ-peptide targeting and AChE/BChE inhibition. However, the potential of metal complexes to modulate *tau* protein aggregation and MAO levels remains an area of significant research interest and needs further investigation [10].

Among metal complexes, *N*-heterocyclic carbene (NHC) complexes, particularly those bearing gold(I) and silver(I), exhibit a wide range of properties, including potent antimicrobial, anticancer, and anti-inflammatory effects [11,12,13,14].

The versatility of NHC ligands allows for the accurate regulation of the properties of these metal complexes, and the current research continues to explore their potential as promising candidates for the treatment of NDDs. However, to date, little literature data address this field of research, and, to the best of our knowledge, no information concerning the inhibition of MAOs by NHC metal complexes has been reported. Conversely, some results on the ability of these complexes to inhibit AChE have been published. For example, the in silico and in vitro cholinesterase (ChE) inhibitory activity of different benzimidazole- and imidazolidine-based Ag-NHC complexes has been studied, revealing IC_50_ values in the nanomolar range and binding mainly to the catalytic anionic sites of AChE and BChE [15,16,17].

Alongside the study of metal complexes in NDDs, experimental and clinical results have drawn attention to proline-rich polypeptides as potential therapeutic agents for treating AD and other neurodegenerative conditions [18,19]. While promising, further research is crucial to validate these findings and develop effective proline-based treatments.

**Figure 1:**
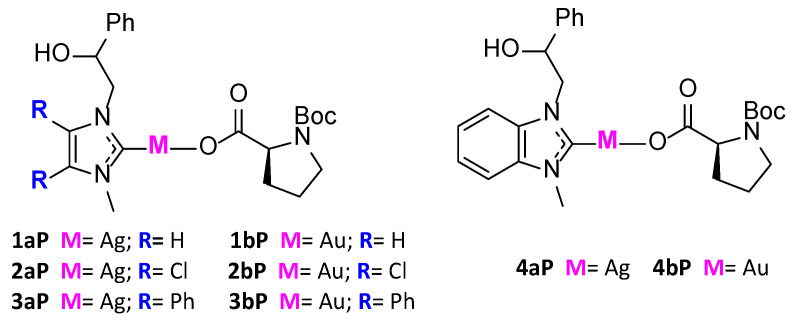
Ag (**1aP**−**4aP**) and Au (**1bP**−**4bP**) complexes stabilized by NHC ligands and bearing carboxylate salt of tert-butyloxycarbonyl (Boc)-*N*-protected proline as anionic ligand.

To deepen the understanding of the protective role of both NHC complexes and proline in neurodegeneration, herein, we designed, synthesized, and biologically characterized, through in silico and in vitro studies, a new series of gold(I) and silver(I) complexes stabilized by NHC ligands and bearing a carboxylate salt of tert-butyloxycarbonyl (Boc)-*N*-protected proline as an anionic ligand (Figure 1, complexes **1aP**−**4aP** and **1bP**−**4bP**) as promising AChEIs and MAOIs. Their antioxidant and anti-inflammatory properties were also evaluated.

## 2. Results and Discussion

### 2.1. Chemistry

**Scheme 1:**
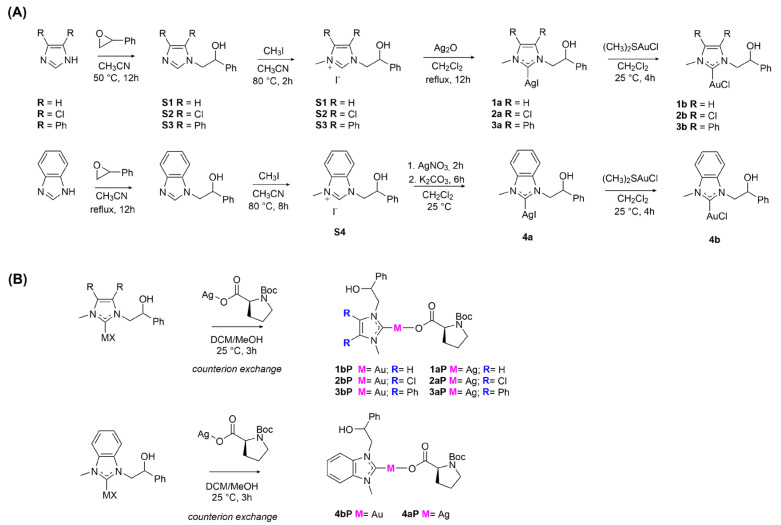
(**A**) Synthesis of precursor metallic complexes **1**–**4a/b**; (**B**) synthesis of co-assembled NHC silver(I) and gold(I) L-proline complexes **1**–**4a/bP**.

The synthetic approach to synthesize the new complexes **1aP**−**4aP** and **1bP**−**4bP** is illustrated in Scheme 1.

They were obtained by starting with the known anticancer active halide complexes **1a**–**4a** and **1b**–**4b**, previously reported by our research group (see Scheme 1A) [20].

The halide ions of the complexes were replaced by *N*-Boc-prolinate-protected anions through a metathesis reaction with *N*-Boc-prolinate-protected silver. Silver halide precipitates and the desired complex were formed according to procedures from the literature (see Scheme 1B) [11,21,22,23].

The co-assembled L-proline complexes were characterized by ^1^H-, ^13^C-NMR, and HRMS. In particular, the appearance of signals in the aliphatic region of the ^1^H-NMR spectra related to the tert-butoxycarbonyl protecting group and the methylene groups of the pyrrolidine ring indicated the presence of an amino acid. Moreover, as observed in the literature [23], an upfield shift of about 5 ppm in the carbenic carbons was detected by ^13^C-NMR, when compared to the parent halogenide complexes. This is evidence of the presence of a stronger σ-donating counteranion, such as a carboxylate group, instead of the halide, which was precipitated as silver iodide during the exchange process.

### 2.2. Cholinesterase-Inhibitory Activity

To assess the potential therapeutic value of the synthesized Ag (**1aP**−**4aP**) and Au (**1bP**−**4bP**) complexes, we first measured their ability to inhibit electric eel acetylcholinesterase (*ee*AChE) and equine butyrylcholinesterase (*eq*BChE). This evaluation followed the established Ellman method [24], using Donepezil as the reference ChE inhibitor. Table 1, reporting the IC_50_ values and SI, shows that the gold complex **4bP,** which had a fused benzene ring at the carbenic ring, was the most potent ChE inhibitor, being more selective toward *eq*BChE. In particular, complex **4bP** showed an *ee*AChE IC_50_ value of 3.72 ± 0.44 µM, which was higher than that of Donepezil (IC_50_ = (2.30 ± 0.53) × 10^−2^ µM). In contrast, complex **4bP** was more active than Donepezil against *eq*BChE, showing an IC_50_ value about five times lower (0.45 ± 0.06 and 2.13 ± 0.12 µM for complex **4bP** and Donepezil, respectively). Complex **2bP** was less active against both enzymes than complex **4bP**, exhibiting IC_50_ values of 4.68 ± 0.47 µM for *ee*AChE and 3.14 ± 0.33 µM for *eq*BChE. Thus, the presence of two chlorine atoms at the carbene of complex **2bP** made it less effective than complex **4bP**. Conversely, the gold complex **3bP**, which bore two phenyl groups at the carbene, possessed slightly lower activity than **4bP** and **2bP**, but it was selective toward AChE. Indeed, it inhibited the activity of *ee*AChE with an IC_50_ value of 5.89 ± 0.56, while no inhibitory activity was recorded for *eq*BChE, at least until reaching a concentration of 25 µM. The absence of substituents at the carbenic ring, as in complex **1bP**, led to a drastic loss of inhibitory activity against *ee*AChE compared with that of the other gold-based complexes (IC_50_ = 18.37 ± 1.43 µM).

All the Ag-based complexes were less active when compared with Au-based analogues. The most active Ag-based complex was **4aP**, in which the carbene ring was fused with a benzene scaffold, with IC_50_ values of 13.95 ± 0.72 µM for *ee*AChE and 5.19 ± 0.95 µM for *eq*BChE. Complexes **2aP** and **3aP**, with two chloride groups or two phenyl groups at the carbene ring, acted as selective *eq*BChE inhibitors with IC_50_ values of 18.24 ± 2.4 µM and 17.38 ± 0.98 µM, respectively. Finally, the absence of substituents at the carbenic ring, as in complex **1aP**, resulted in a total loss of inhibitory activity against both *ee*AChE and *eq*BChE at concentrations of up to 25 µM. Collectively, these outcomes suggest a higher selectivity of the Ag-based complexes **1aP**−**4aP** toward *eq*BChE, with IC_50_ values different to those of other NHC-Ag complexes reported in the literature showing higher selectivity toward AChE [17] or BChE [25] or that were non-selective [26], probably due to the presence of different and bulkier substituents at the carbene ring and the lack of a ProBoc scaffold. Conversely, to the best of our knowledge, less is still known about NHC-Au complexes.

In conclusion, gold-based complexes were better *ee*AChE and *eq*BChE inhibitors than silver-based complexes. The chemical structures of complexes **4bP** and **3bP** may represent a good starting point to develop selective AChE and BChE inhibitors.

### 2.3. MAO-A/B-Inhibitory Activity

Neurodegenerative diseases are often accompanied by a decline in multiple neurotransmitter systems, leading to cognitive and motor deficits. Both ChEs and MAOs are enzymes involved in neurotransmitter metabolism; therefore, targeting both enzymes can have a synergistic effect addressing multiple aspects of these diseases.

Altered levels of MAO-A/B are associated with the degeneration of neurotransmitter biogenic amines such as serotonin, noradrenaline, and dopamine and are implicated in several diseases, such as depression, cancer, PD, and AD [27]. With this in mind, we explored the potential of these complexes in inhibiting human MAO (*h*MAO) isoforms. Clorgyline and (R)-(–)-Deprenyl were used as reference inhibitors for *h*MAO-A and B, respectively. The obtained results are presented in Table 2.

All the gold complexes exhibited higher *h*MAO-inhibitory activity than the related silver analogues. Notably, all the gold-based complexes displayed a higher inhibition potency against *h*MAO-A. Specifically, complexes **4bP** and **2bP** emerged as the most active *h*MAO-A inhibitors. Complex **4bP**, bearing a benzene ring fused to the carbene, demonstrated, approximately, a 10-fold-higher *h*MAO-A-inhibitory activity than complex **2bP**, which bore two chlorine atoms at the carbene ring (the IC_50_ values for complexes **4bP** and **2bP** for *h*MAO-A were (7.13 ± 0.97) × 10^−2^ and 0.72 ± 0.02 µM, respectively). The activity of both complexes against *h*MAO-B was similar, with IC_50_ values of 2.10 ± 0.20 µM and 1.94 ± 0.09 µM for complexes **4bP** and **2bP**, respectively. The remaining gold complexes, **1bP** and **3bP**, showed lower and similar activity against both enzymes. Thus, the presence of either two hydrogen atoms (complex **1bP**) or two phenyl groups (complex **3bP**) at the carbene ring reduced the inhibitory activity against *h*MAOs. Regarding the silver complexes, the presence of a benzene scaffold fused with the carbene core in complex **4aP** resulted in the highest inhibitory activity against *h*MAO-A (IC_50_ = 3.32 ± 0.15 µM). Conversely, complex **2aP**, with two chlorine atoms in the carbene core, exhibited greater selectivity towards *h*MAO-B (IC_50_ = 9.82 ± 0.84 µM). Finally, consistent with the trend observed for gold complexes, the presence of two hydrogen atoms or two phenyl groups at the carbene core made complexes **1aP** and **3aP** completely inactive against both enzymes at concentrations of up to 25 µM. These findings suggest that the nature of the substituents in the carbene core plays a critical role in modulating the inhibitory activity of metal complexes against *h*MAO-A and *h*MAO-B. Moreover, the inhibitory activity against *h*MAO-A and -B mirrored that observed against ChEs. The recorded trend highlights the potential of NHC-Au and Ag complexes as promising starts for the development of MAO inhibitors to be further exploited for therapeutic applications, and, most importantly, the latter outcomes represent the first report that fills the gaps in the scientific literature data. Indeed, to date, many compounds bearing different chemical scaffolds have been reported to inhibit MAO isoforms, but they are not structurally related to the present series [7,28,29].

### 2.4. Anti-Inflammatory Activity

A growing amount of research highlights the role of multiple inflammatory pathways in the development and progression of different NDDs, suggesting that chronic neuroinflammation could significantly amplify disease progression. Thus, regulating the neuroinflammatory response could decrease the production of neurotoxic substances and potentially lead to improved clinical outcomes [30].

**Figure 2:**
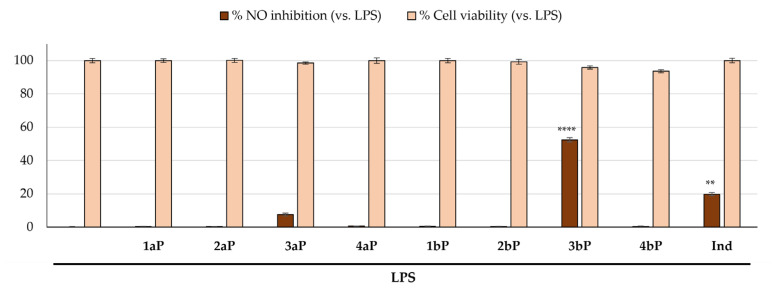
Brown histograms: Anti-inflammatory activity in terms of NO production inhibition (%), measured in vitro using RAW 264.7 murine macrophages. NO production was induced by LPS (1 µg/mL). Data were plotted as % NO inhibition with respect to inhibition with LPS-only treatment. Salmon histograms: MTT assays performed in same experimental conditions in order to verify effect on RAW 264.7 cells’ viability of Indomethacin and tested complexes. Data were plotted as % of cell viability with respect to viability with LPS-only treatment (1 µg/mL). Indomethacin (Ind) was used at concentration of 25 µM, while all other complexes were used at 5 µM, for 24 h. Bars ± SDs are shown (*n* = 3). ** *p* < 0.01 and **** *p* < 0.0001 for complex **3bP** and Ind vs. LPS only; not significant (n.s.) where not indicated.

For this reason, the newly synthesized gold and silver complexes were tested for their potential anti-inflammatory activity, particularly their inducible nitric oxide synthase (iNOS)-inhibitory activity, by means of a Griess-based assay in lipopolysaccharide (LPS)-stimulated murine macrophages, RAW 264.7. As a positive control, the nonsteroidal anti-inflammatory drug (NSAID) Indomethacin (Ind) was used [31]. The RAW 264.7 cell viability after exposure to our complexes and the IC_50_ values for iNOS were calculated and are reported in Table 3. First, as can be observed in Figure 2, amongst the tested complexes, only complex **3bP** produced a great and significant inhibition of NO production of about 52% in LPS-stimulated RAW 264.7 cells at a concentration of 5 µM. For the **3bP** complex, the observed decrease in NO production with respect to that achieved with the LPS-only treatment (%) was statistically significant (*p* < 0.0001). At the same concentration, the silver-based analogue **3aP** inhibited the NO production by approximately 8%. The obtained data demonstrate that both gold and the two phenyl groups at the carbene ring are important structural features required to obtain compounds able to inhibit iNOS. In fact, as can be seen from Table 3, the IC_50_ values related to the inhibition of NO production were 4.88 µM and 30.88 µM for complexes **3bP** and **3aP**, respectively. Furthermore, both complexes showed no toxicity effects on RAW 264.7 cells, as is evident from the cell viability IC_50_ values calculated for the RAW 264.7 cells (20.27 ± 1.26 µM and >50 µM for complexes **3bP** and **3aP**, respectively).

It should be noticed that Ind produced an NO inhibition of about 20% at 25 µM, making it 2.6 times less potent at a concentration five-fold higher compared to complex **3bP**. Anti-inflammatory activity comparable to that of complex **3aP** was also observed for complex **2bP**, which showed a similar IC_50_ value for NO production of 32.31 ± 1.82 µM, without exhibiting toxicity towards murine macrophages up to a concentration of 50 µM (see Table 3). Moreover, complex **4bP** possessed a good IC_50_ value for NO inhibition of 13.05 ± 0.92 µM, but it was proved to be toxic against RAW 264.7 cells (IC_50_ for cell viability = 12.12 ± 1.03 µM). The toxicity of complex **4bP** effectively invalidated the NO inhibition data, as the observed reduction in NO was simply a consequence of cellular death. All the other complexes did not exhibit any anti-inflammatory activity at concentrations up to 50 µM under the employed experimental conditions. The data strongly suggest that the presence of gold and the two phenyl groups in the carbene core are key determinants of iNOS inhibition activity in this series of complexes. Contrarily, the concurrent presence of gold and a benzene ring fused with the carbene core strongly influenced the toxicity of the complexes for the murine macrophage RAW 264.7.

### 2.5. Antioxidant Activity

**Figure 3:**
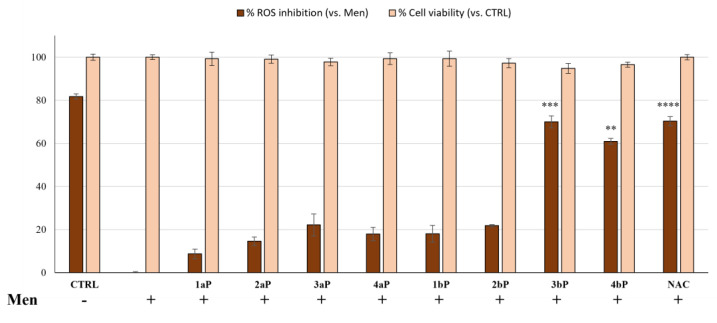
Brown histograms: Antioxidant activity in terms of inhibition of intracellular ROS production (%), measured in vitro using 3T3-L1 murine fibroblasts. ROS production was induced by menadione (Men, 25 µM, 15 min). Data were plotted as % ROS inhibition with respect to inhibition with Men-only treatment. Salmon histograms: MTT assays were performed in order to verify effect on 3T3-L1 cells’ viability of *N*-acetyl cysteine (NAC) and tested complexes. Data were plotted as % of cell viability with respect to viability with CTRL-only treatment (vehicle only). NAC was used at concentration of 20 mM, while all other complexes were used at 10 µM, except for **3bP,** used at 1 µM, for 24 h. Bars ± SDs are shown (*n* = 3). ** *p* < 0.01, *** *p* < 0.001, and **** *p* < 0.0001 for complexes **4bP and 3bP** and NAC vs. only Men; other complexes were not significant (n.s.).

Considering that the exacerbation of intracellular oxidative stress has been implicated in several diseases, including NDDs, in this context, we evaluated the potential ROS scavenging activity of Ag (**1aP**−**4aP**) and Au (**1bP**−**4bP**) complexes in 3T3-L1 murine fibroblasts using a dihydro-2′,7′-dichlorofluorescein diacetate (H_2_DCF-DA) fluorescent probe. Menadione (Men, 25 µM, 15 min) was used to induce ROS production within the cells (Figure 3, brown histograms). *N*-acetyl-cysteine (NAC), a powerful antioxidant, was used as a positive control. The lack of cytotoxicity for all the compounds used in the antioxidant assay at 1 µM (compound **3bP**) or 10 µM (compounds **1aP**–**4aP**, **1bP**, **2bP**, and **4bP**) was ascertained using an MTT test performed under the same experimental conditions (Figure 3, salmon histograms). Only the concentrations that did not significantly affect the cell viability (around 95–99%) were chosen. The best antioxidant complexes were **4bP** and **3bP**, which reduced the ROS production to approximately 60 and 70% at 10 µM and 1 µM, respectively, compared to the Men treatment alone. These activities proved to be highly interesting, considering that NAC reduced the ROS formation to approximately 70%, but only at a concentration of 20 mM. The other complexes decreased the ROS generation by approximately 10–20%. These results clearly demonstrate that all the gold-based complexes showed better in vitro antioxidant capacities compared to the silver analogues. Among the gold-based complexes, the presence of the two phenyl groups as substituents in the carbene core made complex **3bP** the best antioxidant, even at a concentration of 1 µM. It should be noted that complex **3bP** also exhibited the best anti-inflammatory activity amongst all the considered complexes. Furthermore, the benzene scaffold fused with the carbene ring allowed complex **4bP** to inhibit ROS production, although to a lesser extent. Conversely, the presence of two chlorine atoms or the absence of substituents produced a marked reduction in the antioxidant activity, as was evident for the gold complexes **2bP** and **1bP**.

### 2.6. Docking Studies

Molecular docking simulations were performed to investigate the molecular recognition profiles of a series of gold and silver complexes for *h*ChEs, *h*MAOs, and iNOS. The theoretical binding energies were calculated as described in the Experimental Section. The predicted binding energies did not show a direct correlation with the experimentally determined IC_50_ values. This divergence may be attributed to several factors, including the limitations inherent to docking methodologies, the differential activity of enantiomers, the intrinsic limitations of the molecular mechanics energy evaluation of metal complexes, and the influence of solvent effects that were not fully captured in the simulations. Based on the experimental data, attention was focused on the complexes most active against the specified targets mentioned (**2aP**, **2bP**, **4aP,** and **4bP** for *h*ChEs and *h*MAOs and **3aP** and **3bP** for iNOS; Tables S1, S2, and S3).

**Figure 4:**
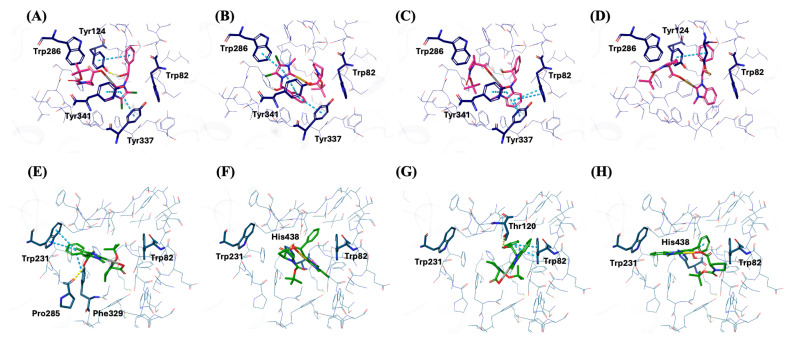
Docking poses of the best-scoring enantiomers of compounds **2aP**, **2bP**, **4aP,** and **4bP** in *h*AChE (**A**–**D**) and *h*BChE (**E**–**H**). The ligands are represented as pink or green sticks and the residues involved in the interaction as violet or blue sticks. (**A**) **(*S*)**–**2aP**; (**B**) (***R*)**–**2bP**; (**C**) **(*S*)**–**4aP**; (**D**) **(*S*)**–**4bP;** (**E**) **(*S*)**–**2aP**; (**F**) **(*R*)**–**2bP**; (**G**) **(*R*)**–**4aP**; (**H**) **(*S*)**–**4bP**.

In the case of *h*AChE, the docking results indicated that both **4aP** and **4bP** oriented the benzimidazole moiety toward the catalytic active site (CAS). Compound **4aP** exhibited π–π stacking interactions with the key residues Trp82, Tyr337, and Tyr341. Similarly, **4bP** formed a hydrogen bond with Tyr124 and π–π stacking with Trp82, His447, and Tyr124. For **2aP**, the imidazole ring was directed toward the CAS, establishing π–π stacking and hydrogen bonds with Tyr341, Tyr337, and Tyr124. Conversely, **2bP** adopted a flipped orientation toward the peripheral anionic site (PAS), where it interacted with Tyr341, Tyr124, and Trp286. The evaluated complexes also displayed favourable binding conformations within the *h*BChE active site. **(*R*)**−**4aP** formed hydrogen bonds and π–π stacking interactions with Thr120 and Trp82, while **(*S*)**−**4aP** engaged Trp231, Phe329, and His438 through π–π interactions. Compound **4bP** showed enantiomer-dependent binding modes: **(*S*)**−**4bP** primarily interacted with His438, whereas **(*R*)**−**4bP** engaged Trp321 and Phe329. Regarding **2aP**, the (*R*)-enantiomer established hydrogen bonds and π–π stacking with Phe329 and Leu286, while the (*S*)-enantiomer interacted with Trp231, Phe329, and Pro285. **(*S*)**−**2bP** engaged in π–π stacking with Phe329, whereas **(*R*)**−**2bP** adopted a flipped pose, establishing hydrogen and halogen bonds with His438 (Figure 4).

**Figure 5:**
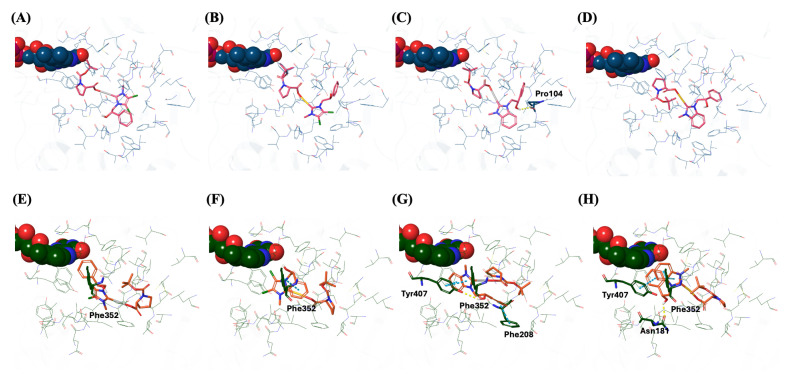
Docking poses of the best-scoring enantiomers of compounds **2aP**, **2bP**, **4aP,** and **4bP** in *h*MAO-A (**A**–**D**) and *h*MAO-B (**E**–**H**). The ligands are represented as pink or orange sticks and the residues involved in the interaction as blue or green sticks. (**A**) **(*S*)**–**2aP**; (**B**) **(*R*)**–**2bP**; (**C**) **(*S*)**–**4aP**; (**D**) **(*S*)**–**4bP;** (**E**) **(*S*)**–**2aP**; (**F**) **(*S*)**–**2bP**; (**G**) **(*S*)**–**4aP**; (**H**) **(*R*)**–**4bP**.

Docking simulations for *h*MAO-A revealed that the benzimidazole moiety of both **4aP** and **4bP** derivatives was oriented toward the flavin adenine dinucleotide (FAD) cofactor. Specifically, **4aP** engaged in π–π stacking interactions with Tyr407 and Tyr444. The (*S*)-enantiomer further established additional π–π stacking and hydrogen bonds with Phe208 and Tyr407. In the case of **4bP**, the (*S*)-enantiomer mainly established van der Waals interactions, whereas the (*R*)-enantiomer formed π–π stacking interactions with Phe352 and Tyr407, along with a hydrogen bond involving Asn181. For **2aP** and **2bP**, the imidazole ring was directed toward the FAD cofactor. An exception was observed with **(*R*)**–**2bP**, which adopted a flipped orientation. The primary interactions were van der Waals interactions or, in some cases, π–π stacking with Phe352 for **(*R*)**–**2bP** and with Tyr407 for the (*S*)-enantiomer. In *h*MAO-B, the docking results indicated that the benzimidazole group of **4aP** and **4bP** derivatives pointed toward the entrance of the catalytic cavity. **4aP** showed enantiomer-specific interactions: the (*R*)-enantiomer exhibited π–π stacking with Trp189, and the (*S*)-enantiomer formed hydrogen bonds with Pro108. The imidazole moiety of **2aP** and **2bP** was oriented toward the entrance of the cavity. Notably, only the (*S*)-enantiomer of **2bP** displayed a well-defined interaction pattern, including π–π stacking with Phe168, one halogen bond with Leu64, and one hydrogen bond with Pro102. The remaining derivatives were predominantly stabilized by non-specific van der Waals interactions (Figure 5).

**Figure 6:**
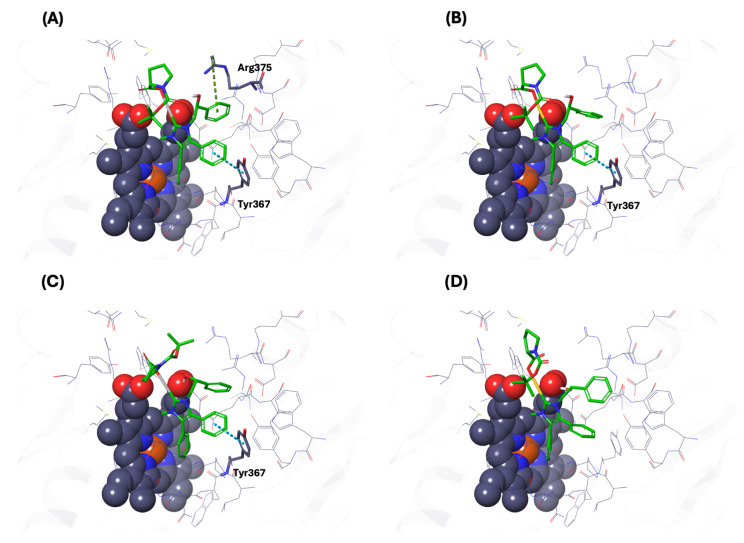
Docking poses of the best-scoring enantiomers of compounds **3aP** and **3bP** in iNOS. The ligands are represented as green sticks and the residues involved in the interaction as blue sticks. (**A**) **(*R*)**–**3aP**; (**B**) **(*R*)**–**3bP**; (**C**) **(*S*)**–**3aP**; (**D**) **(*S*)**–**3bP**.

Docking analyses targeting iNOS revealed that both **3aP** and **3bP** oriented the imidazole moiety toward the interior of the catalytic pocket, establishing key interactions with essential active site residues. In particular, the hydroxyphenylethyl side chain of both compounds engaged in a cation–π interaction with Arg375, while one of the biphenyl–imidazole units formed π–π stacking interactions with Tyr367 (Figure 6).

An in-depth analysis of the metals’ contribution to the activity of our compounds was carried out (Table S6), showing substantial equivalence between Ag and Au. In general, the desolvation energies, as computed by Autodock, revealed that the higher electronegativity of Au leads to a stronger penalty for Au-containing complexes with respect to that for the corresponding Ag analogues. On the other hand, the same property resulted in better electrostatic interaction between the Au-based compounds and the investigated targets.

In summary, the molecular docking studies provided valuable insights into the binding modes and interaction profiles of the most promising gold and silver complexes. The identified interactions with residues known to stabilize enzyme inhibitors support the experimentally observed activities. These findings offer a structural basis for the further optimization and design of more potent derivatives targeting these enzymes.

## 3. Materials and Methods

All the reagents were purchased from Merck Italy (Milan, Italy) and TCI Chemicals (Zwijndrecht, Belgium) and used without any purification. All the solvents were bought from Carlo Erba Reagents srl (Milano, Italy) and were distilled over appropriate drying agents under nitrogen before use. The synthesis of silver(I) and gold(I) complexes was carried out under a nitrogen atmosphere by using Schlenk techniques in the dark. The glassware used was dried in an oven at 120 °C overnight. Deuterated solvents were dried on molecular sieves. ^1^H and ^13^C nuclear magnetic resonance spectra (NMR) were acquired on a Bruker Avance 300 spectrometer (300 MHz for ^1^H; 75 MHz for ^13^C), a Bruker AVANCE 400 spectrometer (400 MHz for ^1^H; 100 MHz for ^13^C), and a Bruker AVANCE 600 spectrometer (600.13 MHz for ^1^H, 150.90 MHz for ^13^C) operating at 298 K. NMR samples were prepared by dissolving about 15 mg of the compound in 0.5 mL of deuterated solvent (Eurisotop Cambridge Isotope Laboratories, Cambridge, UK). The chemical shifts in the ^1^H-NMR and ^13^C-NMR spectra were referenced using the residual proton impurities of the deuterated solvents. The ^1^H-NMR spectra were reported relative to DMSO-d_6_ 1H *δ*H = 2.50 ppm, while the ^13^C-NMR spectra were reported relative to ^13^C *δ*C = 39.52 ppm. The spectrum multiplicities were indicated as follows: singlet (s), doublet (d), triplet (t), multiplet (m), and broad (br). MALDI-MS mass spectra were obtained using a Bruker SolariX XR Fourier transform ion cyclotron resonance mass spectrometer (Bruker Daltonik GmbH, Bremen, Germany) with a 7 T refrigerated actively shielded superconducting magnet (Bruker Biospin, Wissembourg, France). A MALDI ion source (Bruker Daltonik GmbH, Bremen, Germany) was used when examining the samples in the positive ion mode. The mass range was set to m/z 200–3000. The laser power was 28%, and 22 laser shots were utilized for each scan. The mass spectra were calibrated externally using a mix of peptide clusters in the MALDI ionization positive ion mode.

### 3.1. Chemistry

The precursor halide complexes **1**–**4a/b** were synthesized according to the literature procedures reported by us [20,32,33].

#### 3.1.1. Synthesis of Boc-L-Proline Ag(I) Salt

Boc-protected L-proline silver (I) salt was synthesized according to the literature procedures [21]. Boc-L-proline (1.00 g, 4.64 mmol) was dissolved in 0.1 M NaOH (40 mL); to the resulting solution, a silver nitrate (1.7 M in H_2_O) solution was added under stirring. After 15 min, the mixture was partially reduced in volume, and cold Et_2_O was added to obtain an off-white precipitate, which was filtered and dried.

##### Synthesis of Co-Assembled Boc-Proline Silver(I) Complexes **1–4aP** [23]

The Boc amino acid silver(I) NHC complexes **1**–**4aP** were obtained through counterion exchange using silver NHC complexes and Boc-L-Pro silver salt. The silver(I) iodide complexes **1–4a** (0.20 mmol) and Boc-L-Pro silver salt (0.22 mmol) were dissolved in 30 mL of CH_2_Cl_2_/CH_3_OH (6:4) and stirred for 3 h at room temperature, with the exclusion of light. The mixture was filtered to remove the AgI byproduct. The silver NHC-Ag(I)-Boc-L-Pro complex was obtained by removing the solvent at a reduced pressure. The ^1^H-, ^13^C-NMR spectra of complexes **1-4aP** are reported in Figures S1–S8.

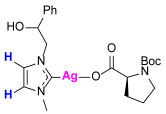



[*N*-methyl, *N′*-(2-hydroxy-2-phenyl) ethyl)-imidazole-2-ylidine silver(I)] Boc-L-Pro (**1aP**, 0.0387 g, 36%).

^1^H-NMR (400 MHz, DMSO-d_6_, ppm) δ: 7.65 (m, 2H, CH_2_NC*H*C*H*N), 7.40–7.29 (m, 5H, C_6_*H_5_*CH(OH)), 4.93 (m, 1H, C_6_H_5_C*H*(OH)CH_2_N), 4.41 (m, 1H, *J*_gem_ = 14.8 Hz, C_6_H_5_CH(OH)C*H_2_*N), 4.24 (m, 1H, *J*_gem_ = 14.8 Hz, C_6_H_5_CH(OH)C*H_2_*N), 3.85–3.78 (overlapping signals, 4H, NC*H_3_* and NC*H*CH_2_ (L-Pro)), 3.30–3.12 (overlapping signals, 2H, NC*H_2_* (L-Pro)), 1.89–1.61 (overlapping signals, 4H, NCHC*H_2_*C*H_2_ *(L-Pro)), 1.34 (s, 9H, C(C*H_3_*)*_3_*).

^13^C-NMR (100 MHz, DMSO-d_6_, ppm) δ: 179.0, 175.0, 163.8, 137.6, 128.7, 126.5, 77.8, 71.0, 61.3, 56.2, 46.4, 36.1, 31.3, 28.6, 24.2, 23.5.

(MALDI-ICR FTMS) m/z: HRMS calculated for C_24_H_28_AgN_4_O_2_^+^: 512.3822, found: 512.3825.

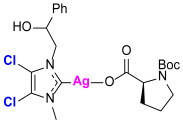



4,5-dichloro [*N*-methyl, *N′*-(2-hydroxy-2-phenyl) ethyl)-imidazole-2-ylidine silver(I)] Boc-L-Pro (**2aP**, 0.0756 g, 64%).

^1^H-NMR (400 MHz, DMSO-d_6_, ppm) δ: 7.48–7.10 (m, 5H, C_6_*H_5_*CH(OH)), 5.29 (br d, 1H, C_6_H_5_CH(O*H*)CH_2_N), 3.86 (m, 1H, C_6_H_5_C*H*(OH)CH_2_N), 3.78 (m, 1H, *J* = 13.5 Hz, 7.5 Hz, C_6_H_5_CH(OH)C*H_2_*N), 3.64 (m, 1H, *J* = 13.5 Hz, 7.5 Hz, C_6_H_5_CH(OH)C*H_2_*N), 3.40–3.19 (overlapping signals, 4H, NC*H_3_* and NC*H*CH_2_ (L-Pro)), 3.30–3.12 (overlapping signals, 2H, NC*H_2_ *(L-Pro)), 1.96–1.61 (overlapping signals, 4H, NCHC*H_2_*C*H_2_ *(L-Pro)), 1.34 (s, 9H, C(C*H_3_*)*_3_*).

^13^C-NMR (100 MHz, DMSO-d_6_, ppm) δ: 180.8, 175.0, 158.1, 154.5, 153.7, 149.0, 135.7, 129.4, 128.7, 128.2, 126.4, 120.3, 77.5, 65.9, 61.1, 55.7, 46.7, 33.4, 31.4, 28.6, 24.2, 23.9.

(MALDI-ICR FTMS) m/z: HRMS calculated for C_24_H_24_AgCl_4_N_4_O_2_^+^: 650.1502, found: 650.1500.

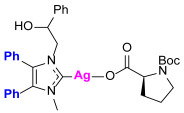



4,5-diphenyl [*N*-methyl, *N′*-(2-hydroxy-2-phenyl) ethyl)-imidazole-2-ylidine silver(I)] Boc-L-Pro (**3aP**, 0.0230 g, 17%).

^1^H-NMR (400 MHz, DMSO-d_6_, ppm) δ: 7.47–7.39 (m, overlapping signals, 9H, Ar-*H*), 7.28–7.26 (m, overlapping signals, 4H, Ar-*H*), 7.09 (dd, 2H, *J* = 7.0, 1.4 Hz, Ar-*H*), 4.73 (br d, 1H, C_6_H_5_CH(O*H*)CH_2_N), 4.22 (br dd, 1H, C_6_H_5_C*H*(OH)CH_2_N), 4.15 (dd, 1H, *J* = 8.5 Hz, 1.4 Hz, C_6_H_5_CH(OH)C*H_2_*N), 3.86 (br dd, 1H, C_6_H_5_CH(OH)C*H_2_*N), 3.80 (s, 3H, NC*H_3_*), 3.32–3.12 (overlapping signals, 3H, NC*H*CH_2_ (L-Pro) and NC*H_2_* (L-Pro)), 1.93–1.62 (overlapping signals, 4H, NCHC*H_2_*C*H_2_ *(L-Pro), 1.34 (s, 9H, C(C*H_3_*)*_3_*).

^13^C-NMR (100 MHz, DMSO-d_6_, ppm) δ: 175.6, 174.6, 154.7, 153.8, 152.1, 141.9, 138.0, 131.8, 131.3, 130.6, 130.5, 129.4, 128.8, 128.3, 126.1, 125.6, 125.4, 77.8, 70.6, 61.4, 61.1, 54.4, 46.6, 46.4, 34.9, 31.2, 30.9, 28.7, 28.5, 24.2, 23.5.

(MALDI-ICR FTMS) m/z: HRMS calculated for C_48_H_44_AgN_4_O_2_^+^: 816.7742, found: 816.7737.

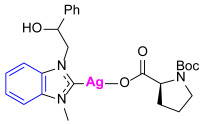



[*N*-methyl, *N′*-(2-hydroxy-2-phenyl) ethyl)-benzoimidazole-2-ylidine silver(I)] Boc-L-Pro (**4aP**, 0.0183 g,16%).

^1^H-NMR (400 MHz, DMSO-d_6_, ppm) δ: 7.98 (d, 2H, *J* = 8.7 Hz, Ar-*H*), 7.62 (2H, m, Ar-*H*), 7.48–7.27 (m, overlapping signals, 5H, Ar-*H*), 5.07 (br d, 1H, C_6_H_5_CH(O*H*)CH_2_N), 4.75 (dd, *J* = 13.8, 2.7 Hz, 1H, C_6_H_5_C*H*(OH)CH_2_N), 4.58 (m, 1H, C_6_H_5_CH(OH)C*H_2_*N), 4.10 (s, 3H, NC*H_3_*), 3.78 (br dd, 1H, C_6_H_5_CH(OH)C*H_2_*N), 3.19–3.12 (overlapping signals, 3H, NC*H*CH_2_ (L-Pro) and NC*H_2_* (L-Pro)), 1.89–1.58 (overlapping signals, 4H, NCHC*H_2_*C*H_2_ *(L-Pro)), 1.31 (s, 9H, C(C*H_3_*)*_3_*).

^13^C-NMR (100 MHz, DMSO-d_6_, ppm) δ: 176.6, 175.4, 163.9, 154.6, 153.7, 152.2, 144.2, 141.7, 139.7, 132.2, 131.8, 128.7, 128.4, 128.2, 126.7, 125.3, 124.2, 114.3, 113.8, 77.6, 70.4, 62.0, 61.6, 53.9, 46.5, 46.3, 33.7, 31.3, 30.9, 28.8, 28.7, 24.2, 23.4.

(MALDI-ICR FTMS) m/z: HRMS calculated for C_32_H_32_AgN_4_O_2_^+^: 612.5022, found: 612.5019.

##### Synthesis of Co-Assembled Boc-Proline Gold(I) Complexes **1–4bP** [23]

The Boc amino acid gold(I) NHC complexes **1**–**4bP** were obtained through counterion exchange using gold NHC complexes and Boc-L-Pro silver salt. The gold(I) chloride complexes **1**–**4b** (0.20 mmol) and Boc-L-Pro silver salt (0.22 mmol) were dissolved in 30 mL of CH_2_Cl_2_/CH_3_OH (6:4) and stirred for 3 h at room temperature, with the exclusion of light. The mixture was filtered to remove the AgCl byproduct. The gold NHC-Au(I)-Boc-L-Pro complex was obtained by removing the solvent at a reduced pressure. The ^1^H-, ^13^C-NMR spectra of complexes **1-4bP** are reported in Figures S9–S16.

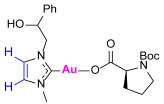



[*N*-methyl, *N′*-(2-hydroxy-2-phenyl) ethyl)-imidazole-2-ylidine gold(I)] Boc-L-Pro (**1bP**, 0.0957 g, 78%).

^1^H-NMR (400 MHz, DMSO-d_6_, ppm) δ: 7.45 (m, 2H, CH_2_NC*H*C*H*N), 7.32–7.21 (m, 5H, C_6_*H_5_*CH(OH)), 4.99 (m, 1H, C_6_H_5_CH(O*H*)CH_2_N), 4.31 (m, 1H, C_6_H_5_C*H*(OH)CH_2_N), 4.04–3.40 (m, overlapping signals, 2H, C_6_H_5_CH(OH)C*H_2_*N), 3.79 (s, 3H, NC*H_3_*), 3.24 (m, 1H, NC*H*CH_2_ (L-Pro)), 2.10–2.02 (overlapping signals, 2H, NC*H_2_* (L-Pro)), 1.82–1.70 (overlapping signals, 4H, NCHC*H_2_*C*H_2_ *(L-Pro)), 1.34 (s, 9H, C(C*H_3_*)*_3_*).

^13^C-NMR (100 MHz, DMSO-d_6_, ppm) δ: 175.6, 171.1, 154.1, 128.9, 126.3, 124.6, 123.6, 79.6, 78.4, 60.2, 46.6, 38.0, 30.9, 28.7, 24.0, 23.3.

(MALDI-ICR FTMS) m/z: HRMS calculated for C_24_H_28_AuN_4_O_2_^+^: 601.4806, found: 601.4809.

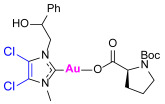



4,5-dichloro [*N*-methyl, *N′*-(2-hydroxy-2-phenyl) ethyl)-imidazole-2-ylidine gold(I)] Boc-L-Pro (**2bP**, 0.0749 g, 55%).

^1^H-NMR (400 MHz, DMSO-d_6_, ppm) δ: 7.43–7.27 (m, 5H, C_6_*H_5_*CH(OH)), 5.95 (br d, 1H, C_6_H_5_CH(O*H*)CH_2_N), 5.00 (m, 1H, C_6_H_5_C*H*(OH)CH_2_N), 4.36 (m, 1H, C_6_H_5_CH(OH)C*H_2_*N), 4.01 (m, 1H, C_6_H_5_CH(OH)C*H_2_*N), 3.88 (m, 3H, NC*H_3_*), 3.79 (m, 1H, NC*H*CH_2_ (L-Pro)), 3.25–3.20 (overlapping signals, 2H, NC*H_2_ *(L-Pro)), 2.07–1.71 (overlapping signals, 4H, NCHC*H_2_*C*H_2_ *(L-Pro)), 1.34 (s, 9H, C(C*H_3_*)*_3_*).

^13^C-NMR (100 MHz, DMSO-d_6_, ppm) δ: 183.4, 176.2, 154.1, 141.8, 129.2, 128.5, 126.7, 118.6, 118.0, 80.0, 79.5, 78.4, 60.4, 57.3, 46.4, 28.6, 24.5, 23.4.

(MALDI-ICR FTMS) m/z: HRMS calculated for C_24_H_24_AuCl_4_N_4_O_2_^+^: 739.2486, found: 739.2487.

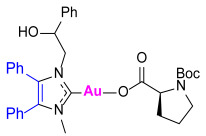



4,5-diphenyl [*N*-methyl, *N′*-(2-hydroxy-2-phenyl) ethyl)-imidazole-2-ylidine gold(I)] Boc-L-Pro (**3bP**, 0.0325 g, 23%).

^1^H-NMR (400 MHz, DMSO-d_6_, ppm) δ: 7.47–7.39 (m, overlapping signals, 9H, Ar-*H*), 7.28–7.26 (m, overlapping signals, 4H, Ar-*H*), 7.09 (dd, 2H, *J* = 7.0, 1.4 Hz, Ar-*H*), 4.73 (br d, 1H, C_6_H_5_CH(O*H*)CH_2_N), 4.22 (br dd, 1H, C_6_H_5_C*H*(OH)CH_2_N), 4.15 (dd, 1H, *J* = 8.5 Hz, 1.4 Hz, C_6_H_5_CH(OH)C*H_2_*N), 3.86 (br dd, 1H, C_6_H_5_CH(OH)C*H_2_*N), 3.80 (s, 3H, NC*H_3_*), 3.32–3.12 (overlapping signals, 3H, NC*H*CH_2_ (L-Pro) and NC*H_2_* (L-Pro)), 1.93–1.62 (overlapping signals, 4H, NCHC*H_2_*C*H_2_ *(L-Pro)), 1.34 (s, 9H, C(C*H_3_*)*_3_*).

^13^C-NMR (100 MHz, DMSO-d_6_, ppm) δ: 175.6, 174.6, 154.7, 153.8, 152.1, 141.9, 138.0, 131.8, 131.3, 130.6, 130.5, 129.4, 128.8, 128.3, 126.1, 125.6, 125.4, 77.8, 70.6, 61.4, 61.1, 54.4, 46.6, 46.4, 34.9, 31.2, 30.9, 28.7, 28.5, 24.2, 23.5.

(MALDI-ICR FTMS) m/z: HRMS calculated for C_48_H_44_AuN_4_O_2_^+^: 905.8726, found: 905.8729.

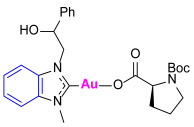



[*N*-methyl, *N′*-(2-hydroxy-2-phenyl) ethyl)-benzoimidazole-2-ylidine gold(I)] Boc-L-Pro (**4bP**, 0.0942 g, 71%).

^1^H-NMR (400 MHz, DMSO-d_6_, ppm) δ: 7.95 (d, 1H, *J* = 6.5 Hz, Ar-*H*), 7.87 (1H, d, *J* = 6.5 Hz, Ar-*H*), 7.57–7.42 (m, overlapping signals, 7H, Ar-*H*), 5.92 (br s, 1H, C_6_H_5_CH(O*H*)CH_2_N), 5.21 (dd, *J* = 14.0, 6.0 Hz, 1H, C_6_H_5_C*H*(OH)CH_2_N), 4.76 (m, 1H, C_6_H_5_CH(OH)C*H_2_*N), 4.18 (d, *J* = 6.9 Hz, 3H, NC*H_3_*), 4.05 (br dd, 1H, C_6_H_5_CH(OH)C*H_2_*N), 3.30–3.24 (overlapping signals, 1H, NC*H*CH_2_ (L-Pro)), 2.10–2.03 (overlapping signals, 2H, NC*H_2_* (L-Pro)), 1.83–1.74 (overlapping signals, 4H, NCHC*H_2_*C*H_2_ *(L-Pro)), 1.38 (s, 9H, C(C*H_3_*)*_3_*).

^13^C-NMR (100 MHz, DMSO-d_6_, ppm) δ: 191.1, 176.6, 154.2, 153.9, 142.5, 134.3, 134.1, 128.8, 128.7, 128.2, 126.7, 124.9, 113.5, 113.4, 112.6, 112.5, 112.2, 78.3, 72.4, 61.0, 60.8, 55.9, 46.6, 46.4, 35.4, 31.3, 30.5, 28.8, 28.7, 24.3, 23.6.

(MALDI-ICR FTMS) m/z: HRMS calculated for C_32_H_32_AuN_4_O_2_^+^: 701.6006, found: 701.60.

### 3.2. Evaluation of Cholinesterase-Inhibitory Activity

The enzyme-inhibiting properties of the Au and Ag complexes against electric eel acetylcholinesterase (*ee*AChE) and equine butyrylcholinesterase (*eq*BChE) were assessed via a modified Ellman assay [34]. Briefly, the enzyme solutions (*ee*AChE: 0.4 U/mL; *eq*BChE: 0.6 U/mL), DTNB (2.14 mM), and the substrates (1.5 mM ATCI and 5.12 mM BTCI) were dissolved and incubated in a 96-well format with the test complexes/standards (concentration range of 0.01–50 µM). The absorbance at 412 nm was monitored over five minutes. A buffer-only control was included. The inhibition potency (IC_50_) was calculated from triplicate dose–response data, presented as the mean ± SD.

### 3.3. Evaluation of Human Monoamine Oxidase (hMAO)-Inhibitory Activity

The inhibitory activity of the gold and silver complexes against *h*MAO-A and *h*MAO-B was evaluated using a previously published protocol [35]. To ensure comparable enzymatic activity, the concentrations of *h*MAO-A (3 ng/mL) and *h*MAO-B (12 ng/mL) were adjusted to achieve a maximum velocity (*V*_max_) of 50 pmol/min for both isoforms. Data analysis was performed using GraphPad PRISM 9 (GraphPad Software^®^, San Diego, CA, USA). IC_50_ values were determined from the dose–response curves and were presented as the mean ± SD of three independent experiments, each conducted in triplicate.

### 3.4. Anti-Inflammatory Activity

To evaluate the anti-inflammatory capacity of the Au and Ag complexes, the nitric oxide (NO) generation in RAW 264.7 murine macrophage cells was quantified using the Griess method [36]. Specifically, the cells were seeded in 48-well plates, treated with varying concentrations of the complexes (range: 1–50 µM; dissolved in DMSO) or Indomethacin (used as a positive control) for a 24 h period, and subsequently stimulated with lipopolysaccharide (LPS, 1 µg/mL). Then, the cell culture medium was combined with an equal volume of Griess reagent at 40 mg/mL (ratio 1:1), and after a 30 min incubation with agitation, the optical density was measured at 540 nm. The levels of NO vs. LPS were calculated from the absorbance measurements using the following formula:

(1) $$I_{NO}\;vs\;LPS\;(\%)=\frac{{ABS}_{LPS}-{ABS}_{Sample}}{{ABS}_{LPS}}*100$$

where ABS_LPS_ represents the average absorbance of cells treated with LPS alone, and ABS_sample_ represents the average absorbance of cells treated with both LPS and the tested complexes or Indomethacin.

3-(4,5-dimethylthiazol-2-yl)-2,5-diphenyl-2H-tetrazolium bromide (MTT) was added to the cells in order to measure the cell viability at 24 h, as previously reported [36]. IC_50_ values were determined from the dose–response curves for both NO inhibition (%) and the cell viability (%) using GraphPad Prism 9 software (GraphPad Software, La Jolla, CA, USA) and were presented as the mean ± SD of three independent experiments, each conducted in triplicate.

### 3.5. Antioxidant Activity

The antioxidant capacity of the Au and Ag complexes was evaluated in 3T3-L1 murine fibroblasts by assessing the intracellular reactive oxygen species (ROS) levels using a DCFH2-DA assay, following a previously established method [36]. The cells were seeded in 96-well plates and incubated with the tested complexes at concentrations of between 0.1 and 20 µM for a 24 h period. Subsequently, ROS generation was triggered by the addition of menadione (25 µM) for 15 min. *N-*acetylcysteine (NAC), a recognized antioxidant, served as a positive control at 20 mM. Then, the cells were loaded with 25 µM DCFH2-DA for 40 min under controlled conditions (dark, 37 °C, 5% CO_2_). At the end of the assay, the fluorescence intensity was measured using a plate reader (λ_ex_ of 485 nm, λ_em_ of 535 nm). The % of ROS production inhibition (I_ROS_) vs. that of the menadione-treated cells was calculated using the following formula:

(2) $$I_{ROS}\;vs\;Men\;(\%)=\frac{F_{Men}-F_{Sample}}{F_{Men}}*100$$

where F_Men_ represents the average fluorescence of the cells treated with menadione alone, and F_sample_ represents the average fluorescence of the cells treated with both menadione and the tested complexes or NAC.

A 3-(4,5-dimethylthiazol-2-yl)-2,5-diphenyl-2H-tetrazolium bromide (MTT) assay was conducted in the same experimental conditions in order to measure the cell viability, as previously reported [36]. IC_50_ values were determined from the cell viability (%) using GraphPad Prism 9 software (GraphPad Software, La Jolla, CA, USA) and were presented as the mean ± SD of three independent experiments, each conducted in triplicate.

### 3.6. Docking Studies

Gold and silver proline complexes were designed using the Maestro graphical user interface [37]. Both the R and S configurations of the carbon 17 stereocenter, derived from the racemic phenyl epoxide synthon (Scheme 1A), were considered. A library of 18 hits was finally obtained. In order to obtain a reasonable starting conformation and a suitable electrostatic partial charge distribution of the ligands (Table S4), the compounds were optimized by means of a Density Functional Theory (DFT) calculation at the B3LYP level, considering a LACV3P** basis set as implemented by Jaguar [38]. All the simulations were carried out in a vacuum environment. A Mulliken electrostatic distribution analysis was used for the next docking simulation.

The Protein Data Bank (PDB) [39] was searched for crystallographic models for *h*AChE, *h*BChE, *h*MAO-A, *h*MAO-B, and NOS2. The PDB entries corresponding to the codes 4EYZ [40], 5NN0 [41], 2Z5X [42], 6FW0 [43], and 3E7M [44] were selected for each mentioned target, respectively. Prior to the docking calculations, the PDB target models underwent suitable optimization, including the removal of water molecules and other co-crystallized chemical entities. Gaistager charges were assigned to the target models by means of Autodock tools ver. 1.5.6 (ADT) [45], and a Mulliken DFT-derived electrostatic distribution was adopted for the ligands. For each target, a 40 × 40 × 40 Å grid centred on the co-crystallized ligand was considered to mimic the binding sites. AutoDock version 4.2.6 [45] was employed with the default parameters. Ten runs of the Lamarckian Genetic Algorithm were applied to explore the ligand–target configurations. Silver and gold atom parameters were obtained from the Autodock Forum [46,47] and implemented in the AutoDock force field (Table S5). The most energy-stable complexes were visually inspected.

### 3.7. Statistical Analysis

The data were analyzed for statistical significance (*p* < 0.05) using a one-way ANOVA followed by Dunnett’s multiple comparison test, performed using GraphPad Prism 9 and in triplicate as indicated in the description of each experiment (*n* = 3). The standard deviations (SD) are shown.

## 4. Conclusions

Despite extensive scientific progress, NDDs continue to impose a significant global burden of disability and morbidity, particularly on the ageing population. The current NDD therapies are often limited by the multifactorial nature of these disorders, and the interconnected presence of neuroinflammation and oxidative stress exacerbates the progression of these diseases. In this context, the exploration of Au- and Ag-NHC metal complexes as potential therapeutic agents for NDDs represents a new and hopeful area. Notably, no information concerning the inhibition of MAOs by Au- and Ag-NHC metal complexes has been reported to date. Our study aimed to contribute to this field by investigating the multi-faceted potential of novel Au- and Ag-NHC complexes bearing a Boc-*N*-protected proline as an anionic ligand, specifically evaluating their antagonism against both ChEs and MAOs, as well as their antioxidant and anti-inflammatory properties. Our findings reveal that Au-based complexes generally exhibit superior inhibitory activity against both *ee*AChE and *eq*BChE and *h*MAO-A and *h*MAO-B compared to their silver analogues. Notably, the Au complex **4bP**, bearing a benzene ring fused to the carbene, demonstrated potent *eq*BChE inhibition and moderate *h*MAO-A inhibition, while complex **3bP**, bearing two phenyl groups on the carbene, acted as a selective *ee*AChE inhibitor and exhibited remarkable anti-inflammatory and antioxidant properties. These results underscore the critical role of the metal and the substituents in the NHC core in modulating the biological activity of these metal complexes. Importantly, our study provides the first evidence of NHC-metal complexes’ inhibition of MAOs and of AChE/BChE inhibition by Au-NHC complexes, filling a significant gap in the current literature. While further in-depth investigations are warranted to fully elucidate their therapeutic potential and safety, the observed multi-target profiles offer a strong rationale for pursuing the development of Au-based NHC-proline complexes as promising novel lead compounds in the fight against NDDs.

## Figures and Tables

**Table 1:** Evaluation of *ee*AChE*/eq*BChE-inhibitory activities of Ag (**1aP**−**4aP**) and Au (**1bP**−**4bP**) complexes and reference inhibitor Donepezil. Values, expressed as µM, are means ± S.D.s of three different experiments performed in triplicate.

IC_50_ (µM)			SI *^a^*
Compounds	*ee*AChE	*eq*BChE	
**Donepezil**	(2.30 ± 0.53) × 10^−2^	2.13 ± 0.12	0.01
**1aP**	>25	>25	-
**2aP**	>25	18.24 ± 2.4	>1.37
**3aP**	>25	17.38 ± 0.98	>1.44
**4aP**	13.95 ± 0.72	5.19 ± 0.95	2.68
**1bP**	18.37 ± 1.43	6.19 ± 0.91	2.97
**2bP**	4.68 ± 0.47	3.14 ± 0.33	1.49
**3bP**	5.89 ± 0.56	>25	>0.24
**4bP**	3.72 ± 0.44	0.45 ± 0.06	8.27

^*a*^ *eq*BChE selectivity index = IC_50_(*ee*AChE)/IC_50_(*eq*BChE).

**Table 2:** Evaluation of *h*MAO-inhibitory activities of Ag (**1aP**−**4aP**) and Au (**1bP**−**4bP**) complexes and reference MAOIs (Clorgyline for *h*MAO-A and (R)-(–)-Deprenyl for *h*MAO-B). Values, expressed as µM, are means ± S.D.s of three different experiments performed in triplicate.

IC_50_ (µM)			SI *^a^*
Compounds	*h*MAO-A	*h*MAO-B	
**Clorgyline**	(2.00 ± 0.17) × 10^−3^	2.44 ± 0.17	8.19 × 10^−4^
**(R)-(–)-Deprenyl**	17.49 ± 2.67	(4.92 ± 0.87) × 10^−2^	356.94
**1aP**	>25	>25	-
**2aP**	15.04 ± 1.02	9.82 ± 0.84	1.53
**3aP**	24.97 ± 2.95	>25	0.99
**4aP**	3.32 ± 0.15	10.40 ± 1.95	0.32
**1bP**	1.32 ± 0.02	3.78 ± 0.16	0.35
**2bP**	0.72 ± 0.02	1.94 ± 0.09	0.37
**3bP**	1.84 ± 0.18	4.75 ± 0.75	0.39
**4bP**	(7.13 ± 0.97) × 10^−2^	2.10 ± 0.20	0.03

*^a^ h*MAO-B selectivity index = IC_50_(*h*MAO-A)/IC_50_(*h*MAO-B).

**Table 3:** IC_50_ values for NO inhibition and the cell viability (%), measured in vitro using RAW 264.7 murine macrophages for the Ag (**1aP**−**4aP**) and Au (**1bP**−**4bP**) complexes and Indomethacin (used as a reference molecule). The values, expressed as µM, are the means ± SDs of three different experiments, performed in triplicate.

IC_50_ (µM)		
Compounds	NO Inhibition	Cell Viability
**Indomethacin**	>50	>50
**1aP**	>50	>50
**2aP**	>50	>50
**3aP**	30.88 ± 1.74	>50
**4aP**	>50	>50
**1bP**	>50	>50
**2bP**	32.31 ± 1.82	>50
**3bP**	4.88 ± 0.58	20.27 ± 1.26
**4bP**	13.05 ± 0.92	12.12 ± 1.03

## Data Availability

Data contained within the article.

## Supplementary Materials

The following supporting information can be downloaded at https://www.mdpi.com/article/10.3390/ijms26136116/s1.
