# Supplementary material for: Novel Au(I)- and Ag(I)-NHC Complexes with N-Boc-Protected Proline as Potential Candidates for Neurodegenerative Disorders
Source: Int J Mol Sci. 2025 Jun 25;26(13):6116. doi: 10.3390/ijms26136116 (PMC12249917; doi:10.3390/ijms26136116)
Supplement: Supplementary file 1 [file ijms-26-06116-s001.zip › ijms-3676076-supplementary.pdf]

Supplementary Materials

Article

Novel Au(I)- and Ag(I)-NHC Complexes with *N*-Boc-Protected Proline as Potential Candidates for Neurodegenerative Disorders

Jessica Ceramella [1,†], Assunta D'Amato [2,†], Francesca Procopio [3], Annaluisa Mariconda [4], Daniel Chavarria [5,*], Domenico Iacopetta [1,*], Francesco Ortuso [3], Pasquale Longo [2], Fernanda Borges [6,7,8,‡] and Maria Stefania Sinicropi [1,‡]

Academic Editor: Cristoforo Comi

Received: 16 May 2025
Revised: 20 June 2025
Accepted: 23 June 2025
Published: 25 June 2025

Citation: Ceramella, J.; D'Amato, A.; Procopio, F.; Mariconda, A.; Chavarria, D.; Iacopetta, D.; Ortuso, F.; Longo, P.; Borges, F.; Sinicropi, M.S. Novel Au(I)- and Ag(I)-NHC Complexes with *N*-Boc-Protected Proline as Potential Candidates for Neurodegenerative Disorders. *Int. J. Mol. Sci.* **2025**, *26*, 6116. https://doi.org/10.3390/ijms26136116

Copyright: © 2025 by the authors. Licensee MDPI, Basel, Switzerland. This article is an open access article distributed under the terms and conditions of the Creative Commons Attribution (CC BY) license (https://creativecommons.org/licenses/by/4.0/).

[1] Department of Pharmacy, Health and Nutritional Sciences, University of Calabria, Via Pietro Bucci, 87036 Arcavacata di Rende, Italy; jessica.ceramella@unical.it (J.C.) s.sinicropi@unical.it (M.S.S.)
[2] Department of Chemistry and Biology "A. Zambelli", University of Salerno, Via Giovanni Paolo II 132, 84084 Fisciano, Italy; asdamato@unisa.it (A.D.); plongo@unisa.it (P.L.)
[3] Dipartimento di Scienze della Salute, Università "Magna Græcia" di Catanzaro, Viale Europa, 88100 Catanzaro, Italy; francesca.procopio001@studenti.unicz.it (F.P.); ortuso@unicz.it (F.O.)
[4] Department of Basic and Applied Sciences (DISBA), University of Basilicata, Via Dell'Ateneo Lucano 10, 85100 Potenza, Italy; annaluisa.mariconda@unibas.it
[5] CIQUP-IMS—Centro de Investigação em Química da Universidade do Porto, Institute of Molecular Sciences, Department of Chemistry and Biochemistry, Faculty of Sciences, University of Porto, Rua do Campo Alegre s/n, 4169-007 Porto, Portugal
[6] MedInUP, Center for Drug Discovery and Innovative Medicines, University of Porto, 4200-319 Porto, Portugal; fborges@fc.up.pt
[7] Department of Chemistry and Biochemistry, Faculty of Sciences, University of Porto, Rua do Campo Alegre s/n, 4169-007 Porto, Portugal
[8] Department of Biomedicine-Pharmacology and Therapeutics Unit, Faculty of Medicine, University of Porto, 4200-319 Porto, Portugal
* Correspondence: daniel.chavarria@fc.up.pt (D.C.); domenico.iacopetta@unical.it (D.I.)
† These authors contributed equally to this work.
‡ Co-senior authors.

Table S1. Comparison among experimentally derived IC_{50} and docking results of silver and gold complexes against *h*AChE and *h*BChE targets

Ligand	Enatiomer	*h*AChE		*h*BChE	
		IC_{50} (μM)	*Computed Binding Energy (Kcal/mol)*	*IC_{50} (μM)*	*Computed Binding Energy (Kcal/mol)*
**2aP**	***(R)***	>25	-9.04	18.2 ± 0.02	-9.20
	(S)		-9.98		-10.85
**2bP**	***(R)***	4.7 ± 0.02	-10.34	3.1 ± 0.02	-10.81
	(S)		-10.22		-10.49
**4aP**	***(R)***	13.9 ± 0.01	-9.87	5.2 ± 0.05	-10.47
	(S)		-9.95		-10.37
**4bP**	***(R)***	3.7 ± 0.02	-9.51	0.45 ± 0.02	-9.62
	(S)		-9.63		-9.84

Table S2. Comparison among experimentally derived IC_{50} and docking results of silver and gold complexes against *h*MAO-A and *h*MAO-B targets

Ligand	Enatiomer	*h*MAOA		*h*MAOB	
		IC_{50} (μM)	*Computed Binding Energy (Kcal/mol)*	*IC_{50} (μM)*	*Computed Binding Energy (Kcal/mol)*
**2aP**	***(R)***	15.0±0.9	-6.48	9.82±0.78	-10.08
	(S)		-7.02		-10.22
**2bP**	***(R)***	$7.4x10^{-1}$±0.016	-6.23	1.94±0.08	-10.77
	(S)		-5.41		-10.83
**4aP**	***(R)***	3.3 ± 0.01	-3.02	10.4 ± 0.03	-10.34
	(S)		-6.56		-10.85
**4bP**	***(R)***	$7.1x10^{-2}$ ± 0.07	-3.50	2.1 ± 0.02	-10.44
	(S)		-5.64		-9.51

Table S3. Comparison among experimentally derived IC_{50} and docking results of silver and gold complexes against iNOS

Ligand	Enatiomer	iNOS	
		IC_{50} (μM)	*Computed Binding Energy (Kcal/mol)*
**3aP**	***(R)***	>50	-9.18
	(S)		-9.94
**3bP**	***(R)***	4.9 ± 0.5	-9.05
	(S)		-10.10

Table S4. Ligands partial atom charge distribution. Hydrogen atoms data are hidden for clarity.

Atom IDs	Ligands							
	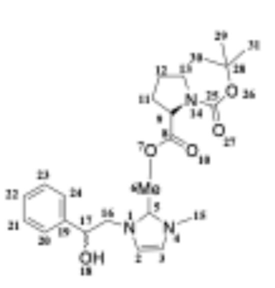 1aP (Me=Ag), 1bP (Me=Au)		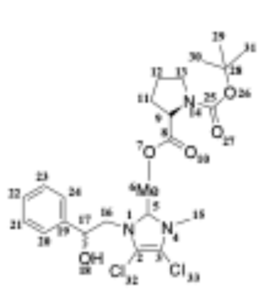 2aP (Me=Ag), 2bP (Me=Au)		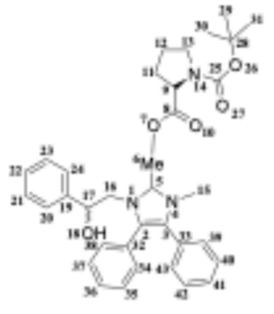 3aP (Me=Ag), 3bP (Me=Au)		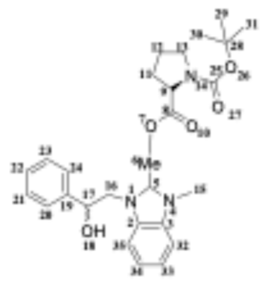 4aP (Me=Ag), 4bP (Me=Au)	
1	-0.33947	-0.31770	-0.30730	-0.30519	-0.34526	-0.34834	-0.38299	-0.37669
2	0.02461	0.03006	0.07622	0.07973	0.11647	0.12592	0.14941	0.15208
3	0.01886	0.01810	0.08098	0.08420	0.11608	0.11985	0.14070	0.14525
4	-0.31767	-0.32356	-0.33980	-0.33065	-0.36887	-0.35874	-0.39567	-0.39427
5	0.14304	-0.12335	0.16025	-0.08549	0.15681	-0.10460	0.13684	-0.11737
6	0.20105	0.46096	0.22296	0.45328	0.20256	0.45963	0.21630	0.47124
7	-0.49328	-0.57464	-0.47247	-0.54804	-0.48769	-0.56612	-0.43546	-0.58229
8	0.40807	0.41723	0.46827	0.48629	0.41716	0.42734	0.44338	0.43818
9	0.02852	0.02761	-0.02976	-0.03605	0.02349	0.02203	-0.03937	-0.03775
10	-0.43557	-0.41085	-0.48719	-0.45686	-0.44634	-0.42243	-0.48813	-0.40007
11	-0.24151	-0.24313	-0.23233	-0.23288	-0.24283	-0.24256	-0.22112	-0.21955
12	-0.24072	-0.24097	-0.26879	-0.26584	-0.23680	-0.24006	-0.27267	-0.27515
13	-0.00692	-0.00775	0.01021	0.01433	-0.01012	-0.00874	0.01182	0.01400
14	-0.43749	-0.43874	-0.42412	-0.42421	-0.43572	-0.43969	-0.41962	-0.42291
15	-0.17607	-0.16820	-0.15374	-0.15406	-0.14923	-0.15299	-0.15440	-0.16265
16	-0.05273	-0.06670	-0.08887	-0.11099	-0.06223	-0.06856	-0.06344	-0.07231
17	0.07418	0.05730	0.06129	0.07872	0.10430	0.10655	0.10132	0.09300
18	-0.39027	-0.39117	-0.39161	-0.38694	-0.39222	-0.39087	-0.41251	-0.40998
19	-0.18596	-0.17400	-0.10931	-0.11686	-0.20493	-0.20808	-0.19930	-0.18791
20	-0.07582	-0.08216	-0.08878	-0.08701	-0.08576	-0.08751	-0.11963	-0.12279
21	-0.09330	-0.09206	-0.09079	-0.09045	-0.09112	-0.09201	-0.10662	-0.10613
22	-0.10768	-0.10811	-0.10847	-0.10909	-0.10974	-0.11038	-0.11269	-0.11213

23	-0.09334	-0.09294	-0.09770	-0.09789	-0.10247	-0.10126	-0.09244	-0.09096
24	-0.06775	-0.06818	-0.10251	-0.10358	-0.09638	-0.08625	-0.08597	-0.08263
25	0.59525	0.59786	0.60066	0.59814	0.59727	0.60002	0.59801	0.60593
26	-0.39187	-0.39105	-0.39622	-0.39697	-0.39190	-0.39258	-0.39375	-0.39155
27	-0.42452	-0.42682	-0.42180	-0.42064	-0.42639	-0.42631	-0.42990	-0.43630
28	0.01078	0.01171	0.00809	0.00376	0.00990	0.01049	0.00721	0.00650
29	-0.25907	-0.26130	-0.26259	-0.26725	-0.26129	-0.26346	-0.26570	-0.26807
30	-0.28671	-0.28647	-0.27555	-0.26633	-0.28020	-0.28180	-0.26930	-0.27025
31	-0.25359	-0.24938	-0.25042	-0.24607	-0.25004	-0.25079	-0.24783	-0.24616
32			0.01306	0.01593	-0.12574	-0.12829	-0.08783	-0.08535
33			0.01655	0.01886	-0.12456	-0.11914	-0.10308	-0.10301
34					-0.07015	-0.06875	-0.10933	-0.10583
35					-0.09185	-0.09328	-0.05423	-0.06421
36					-0.10562	-0.10512		
37					-0.09194	-0.09134		
38					-0.04599	-0.04533		
39					-0.06290	-0.06742		
40					-0.09472	-0.09395		
41					-0.10662	-0.10607		
42					-0.08997	-0.08956		
43					-0.04747	-0.04880		

Table S5. Au and Ag atoms force field parameters adopted for docking simulation.

**Atom Type**	**R_{ii}**	**eps_{ii}**	**vol**	**solpar**	**R_{ij}_hbond**	**esp_{ij}_hb**	**hbond**	**rec_index**	**map_index**	**bond_index**
Au	3.29	0.039	12	-0.0011	0	0	0	-1	-1	4
Ag	3.15	0.036	12	-0.0011	0	0	0	-1	-1	4

where **R_{ii}** = sum of vdW radii of two like atoms (in Angstrom); **eps_{ii}** = vdW well depth (in Kcal/mol); **vol** = atomic solvation volume (in Angstrom^3); **solpar** = atomic solvation parameter; **R_{ij}_hb** = H-bond radius of the heteroatom in contact with a hydrogen (in Angstrom); **eps_{ij}_hb** = well depth of H-bond (in Kcal/mol); **hbond** = integer indicating type of H-bonding atom (0=no H-bond); **rec_index** = initialised to -1, but later on holds count of how many of this atom type are in receptor; **map_index** = initialised to -1, but later on holds the index of the AutoGrid map; **bond_index** = used in AutoDock to detect bonds; see "mdist.h", enum {C,N,O,H,XX,P,S}.

Table S6. Metal contribution on ligands desolvation energy and target electrostatic interaction (in kcal/mol)

Ligand	Metal	Desolvation energy*	Enantiomer	Target	Electrostatic interaction*
**2aP**	Ag	+0.013	(R)	hAChE	-0.19
				hBuChE	-0.11
				hMAO-A	+0.01
				hMAO-B	-0.05
			(S)	hAChE	-0.20
				hBuChE	-0.15
				hMAO-A	+0.01
				hMAO-B	-0.05
**2bP**	Au	+0.032	(R)	hAChE	-0.42
				hBuChE	-0.22
				hMAO-A	-0.10
				hMAO-B	-0.12
			(S)	hAChE	-0.43
				hBuChE	-0.23
				hMAO-A	+0.00
				hMAO-B	-0.11
**3aP**	Ag	+0.019	(R)	iNOS	-0.55
			(S)		-0.52
**3bP**	Au	+0.053	(R)		-1.26
			(S)		-0.43
**4aP**	Ag	+0.014	(R)	hAChE	-0.18
				hBuChE	-0.12
				hMAO-A	0.03
				hMAO-B	-0.05
			(S)	hAChE	-0.26
				hBuChE	-0.12
				hMAO-A	+0.04
				hMAO-B	-0.06
**4bP**	Au	+0.035	(R)	hAChE	-0.41
				hBuChE	-0.23
				hMAO-A	+0.02
				hMAO-B	-0.13
			(S)	hAChE	-0.44
				hBuChE	-0.22
				hMAO-A	+0.02
				hMAO-B	-0.13

^{1}H- and ^{13}C-NMR SPECTRA OF COMPLEXES 1-4aP, 1-4bP

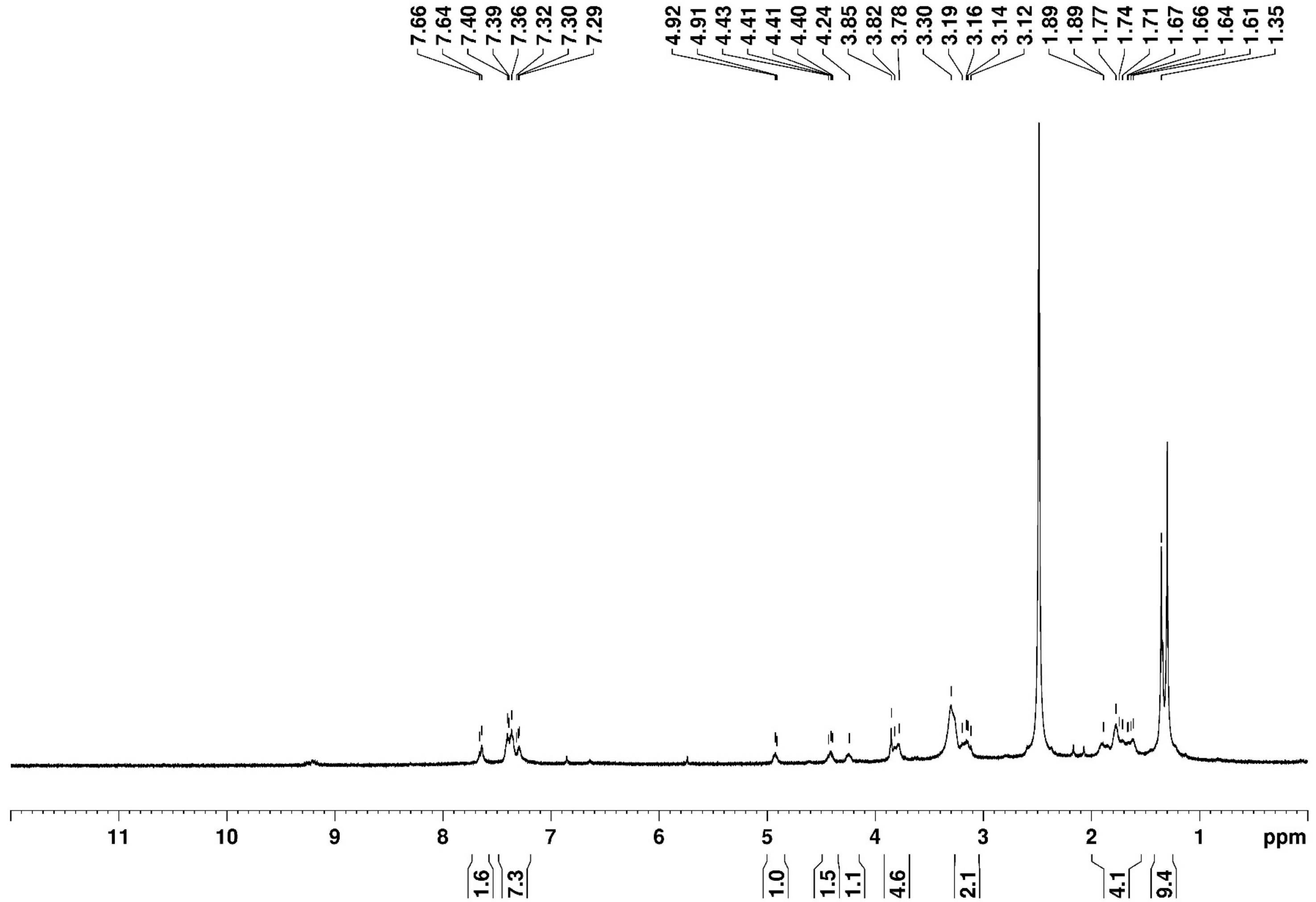

Figure S1. 1aP: ^{1}H NMR (400 MHz, DMSO-d$_6$, 298K)

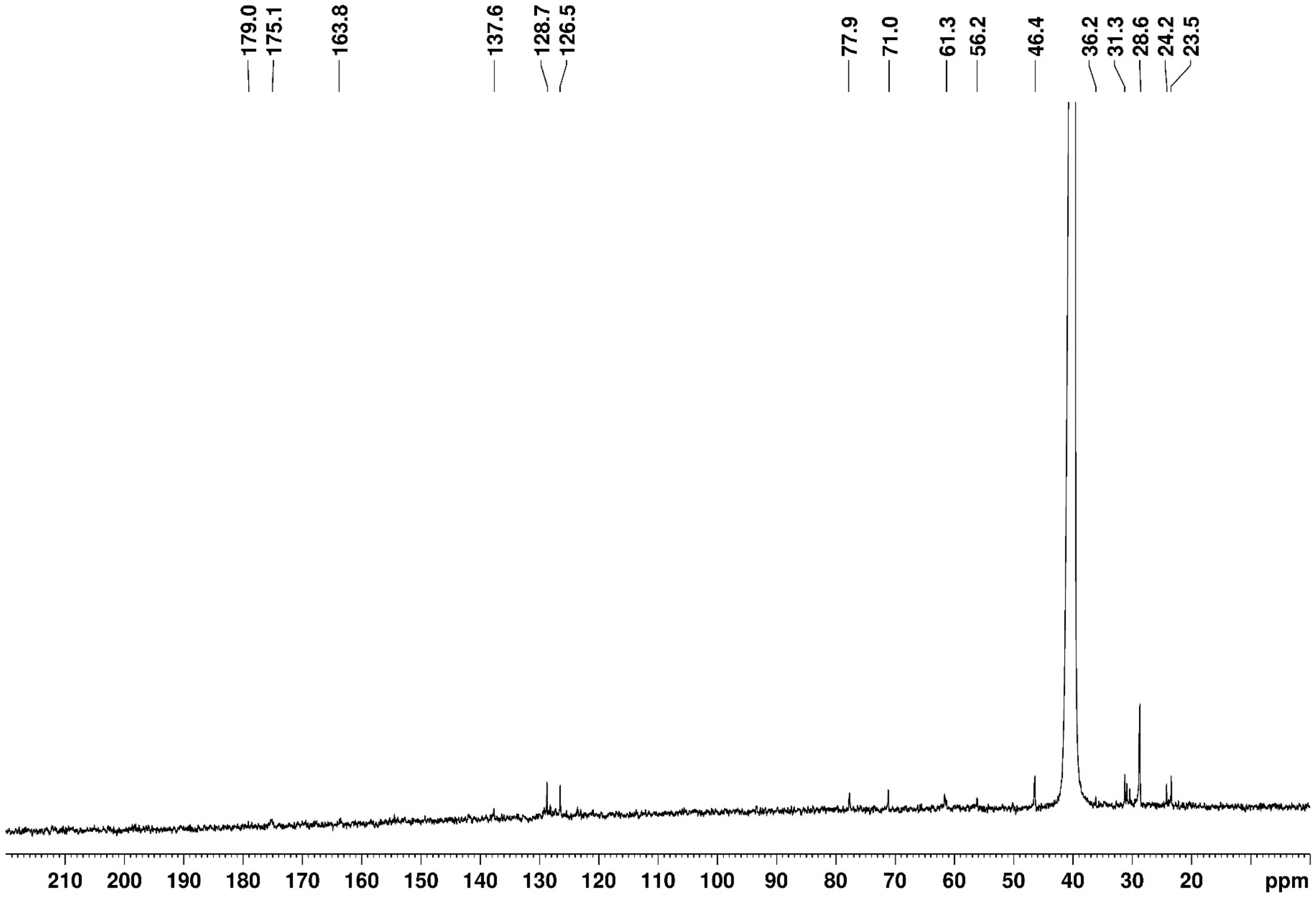

Figure S2. 1aP: ^{13}C NMR (100 MHz, DMSO-d_6, 298K)

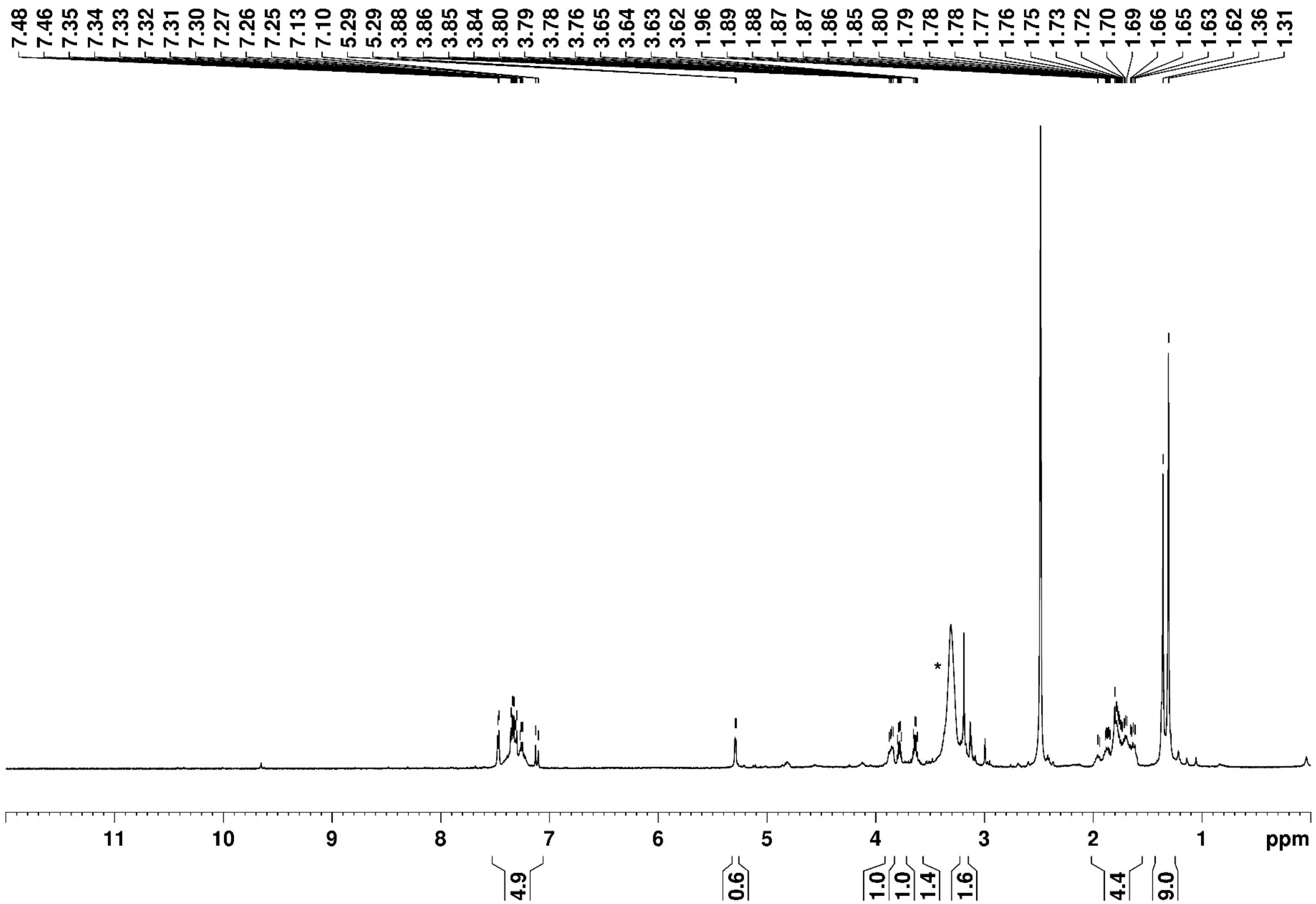

Figure S3. 2aP: ^{1}H NMR (400 MHz, DMSO-d$_6$, 298K)

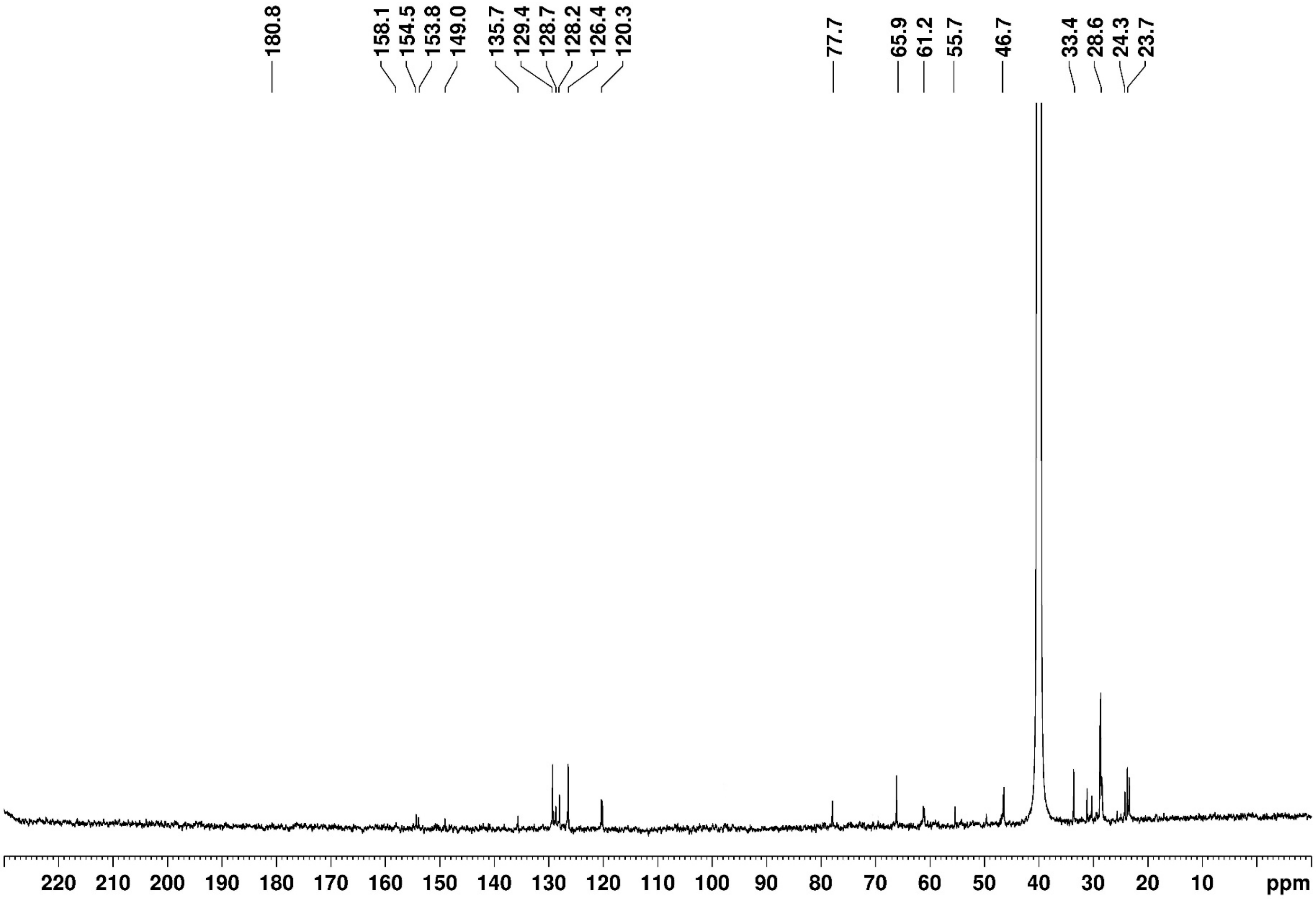

Figure S4. 2aP: ^{13}C NMR (100 MHz, DMSO-d_6, 298K)

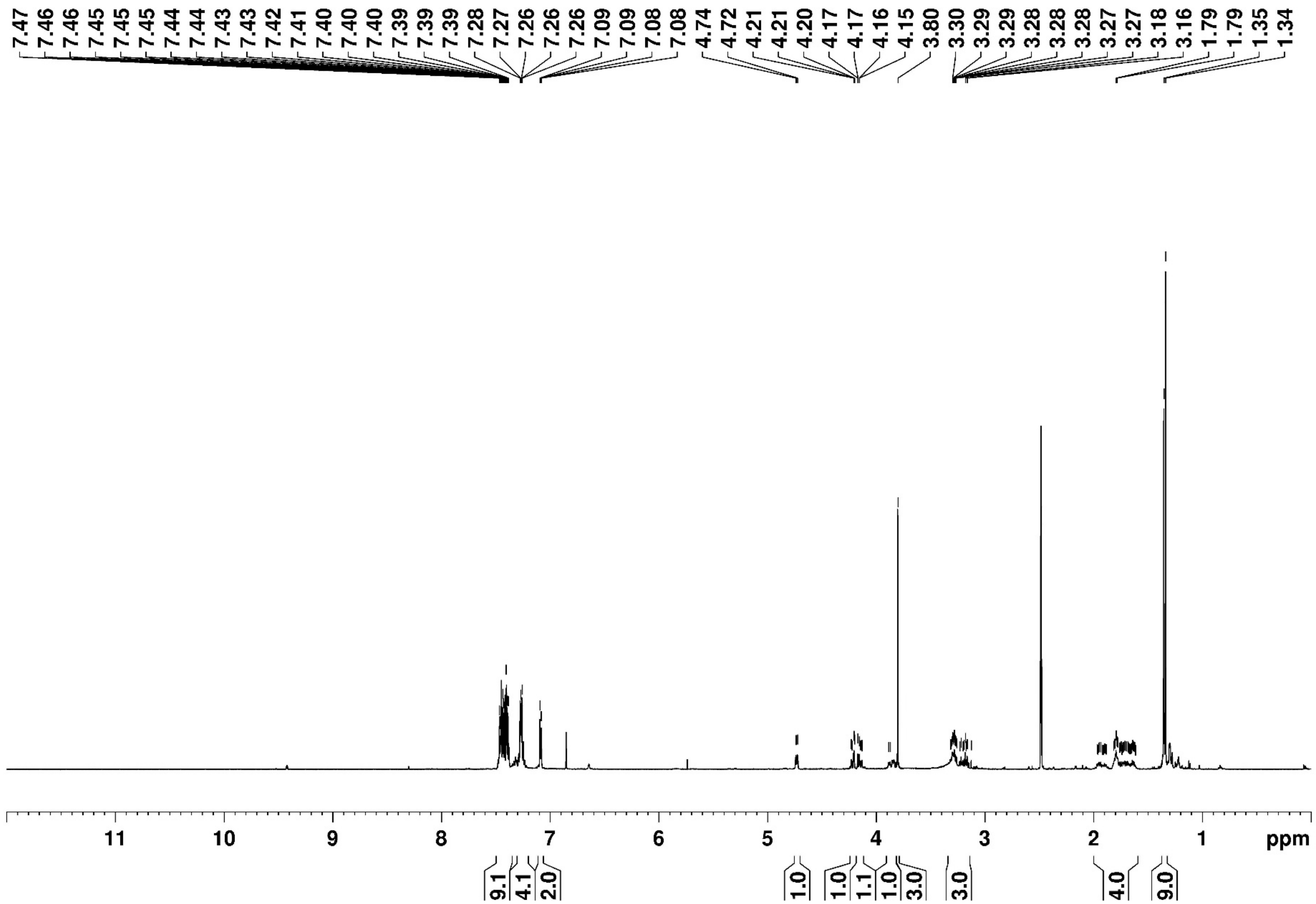

Figure S5. 3aP: ^{1}H NMR (400 MHz, DMSO-d$_6$, 298K)

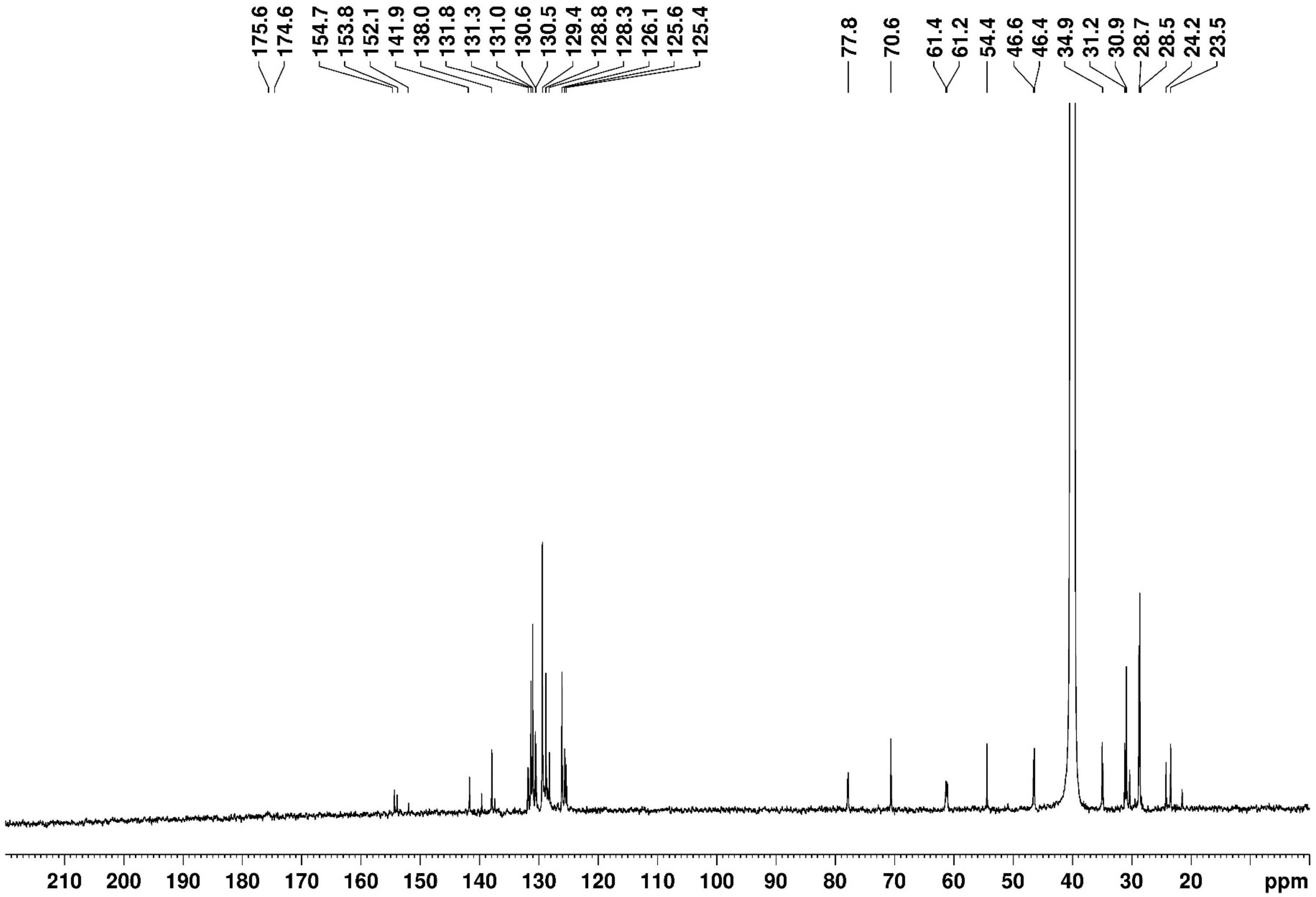

Figure S6. 3aP: ^{13}C NMR (100 MHz, DMSO-d_6, 298K)

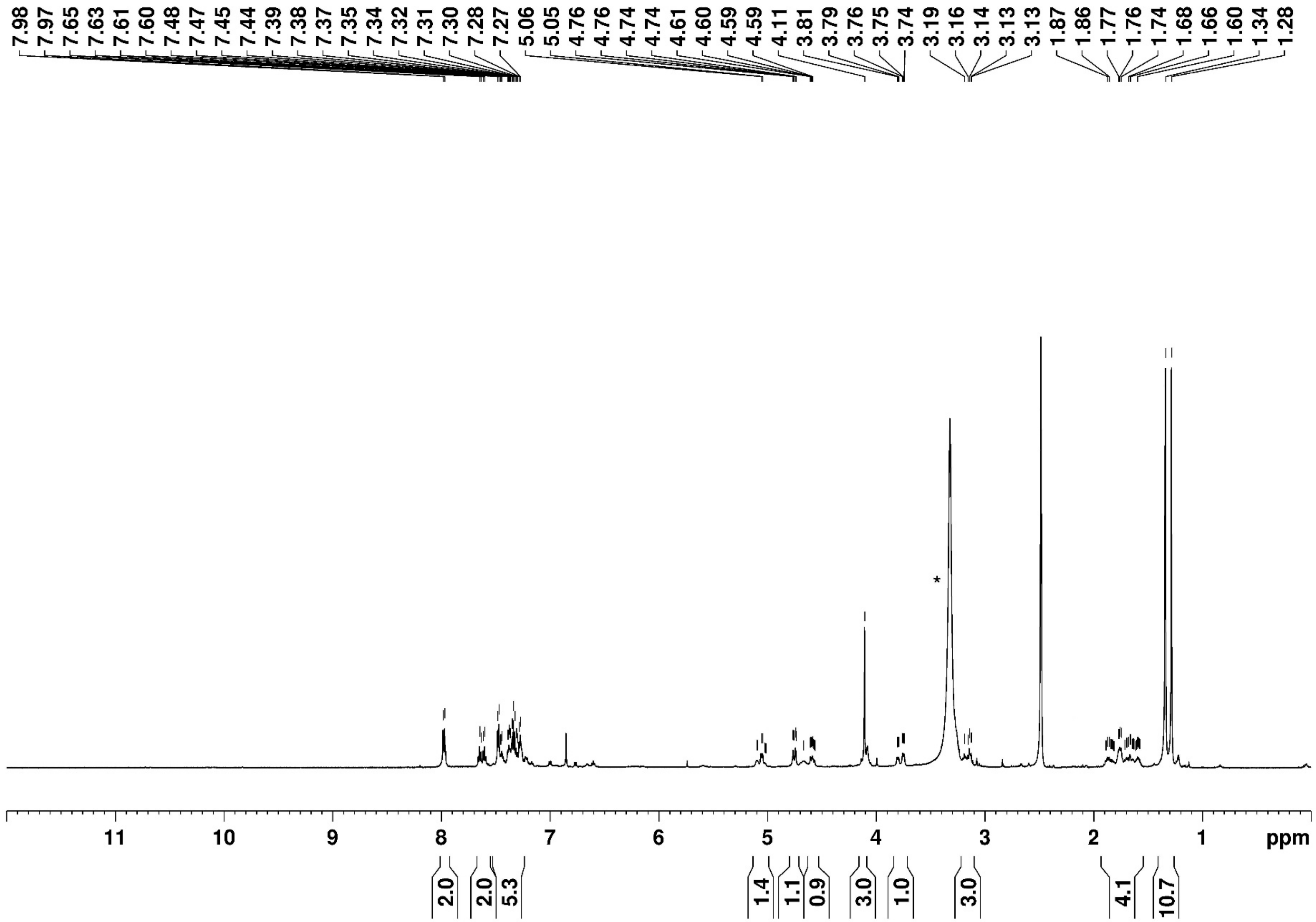

Figure S7. 4aP: ^{1}H NMR (400 MHz, DMSO-d$_6$, 298K)

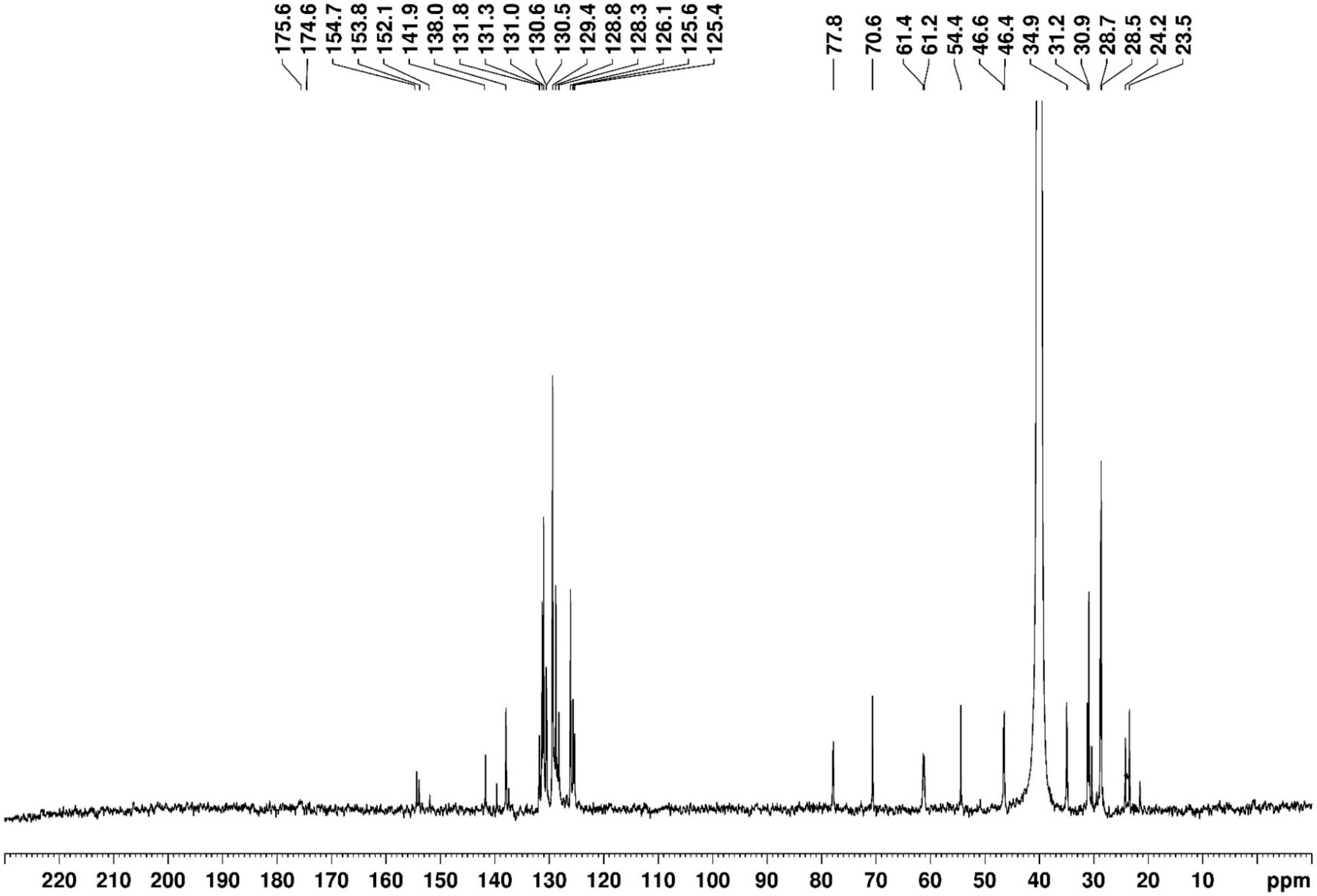

Figure S8. 4aP: ^{13}C NMR (100 MHz, DMSO-d_6, 298K)

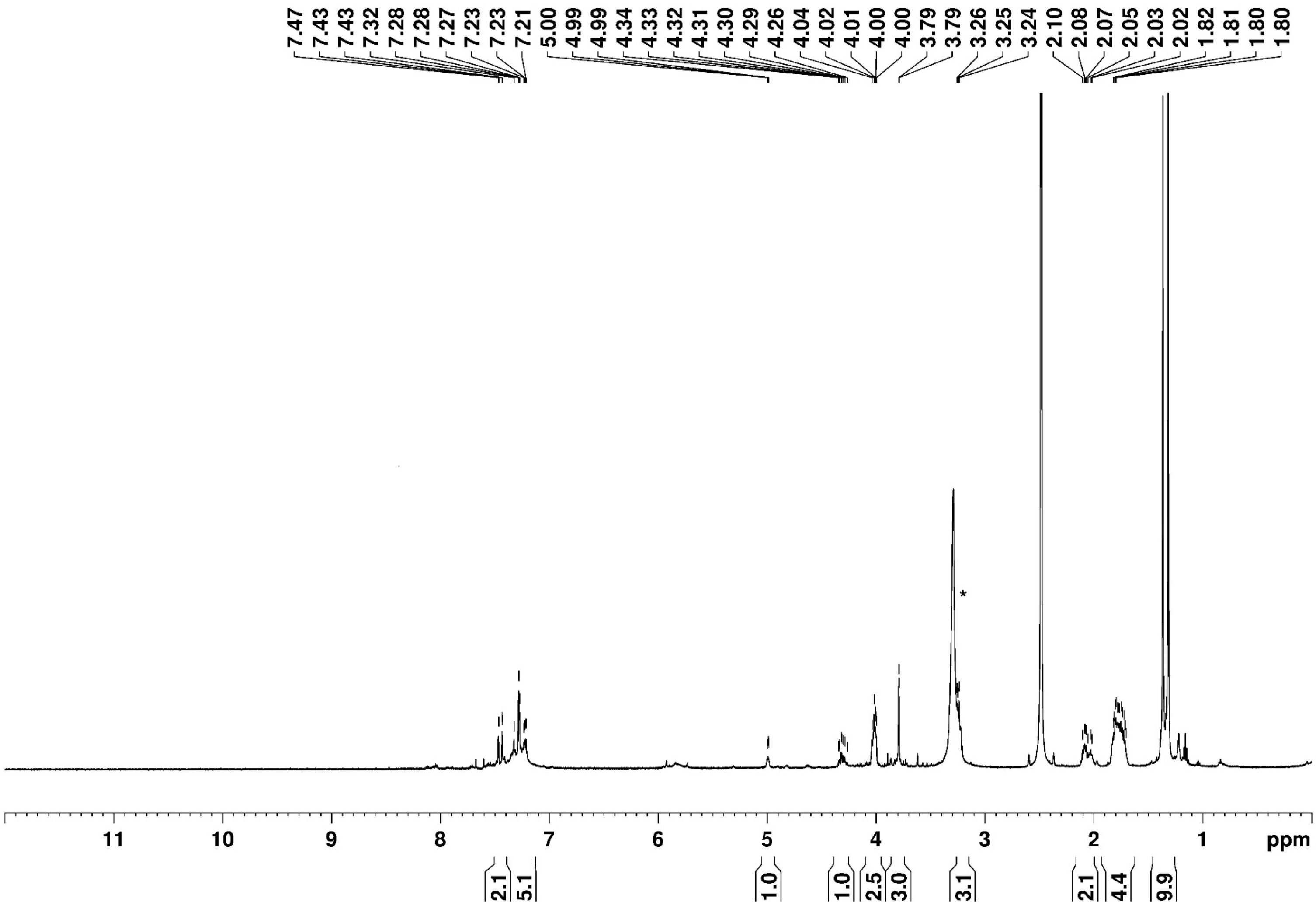

Figure S9. 1bP: ^{1}H NMR (400 MHz, DMSO-d$_6$, 298K)

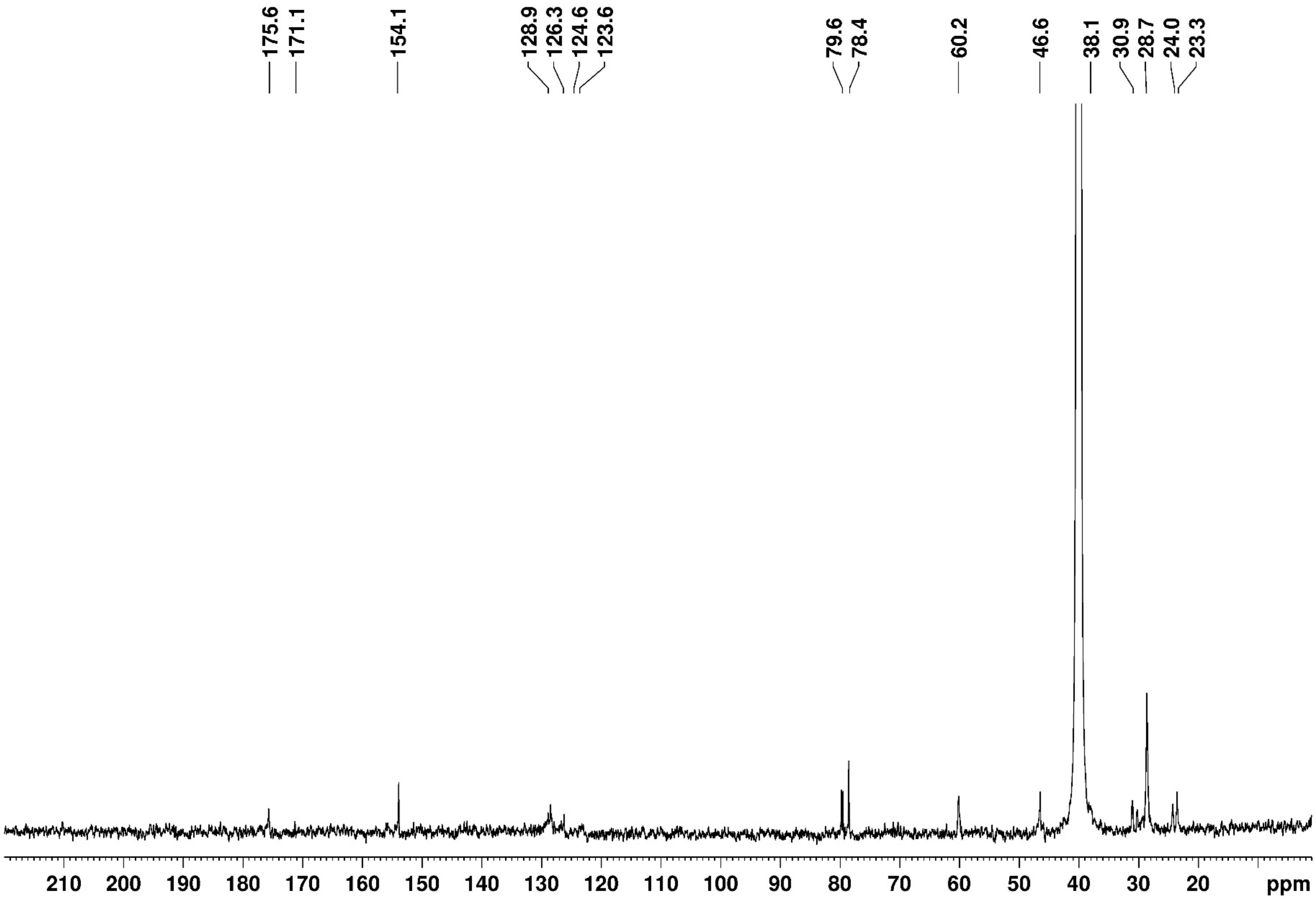

Figure S10. 1bP: ^{13}C NMR (100 MHz, DMSO-d_6, 298K)

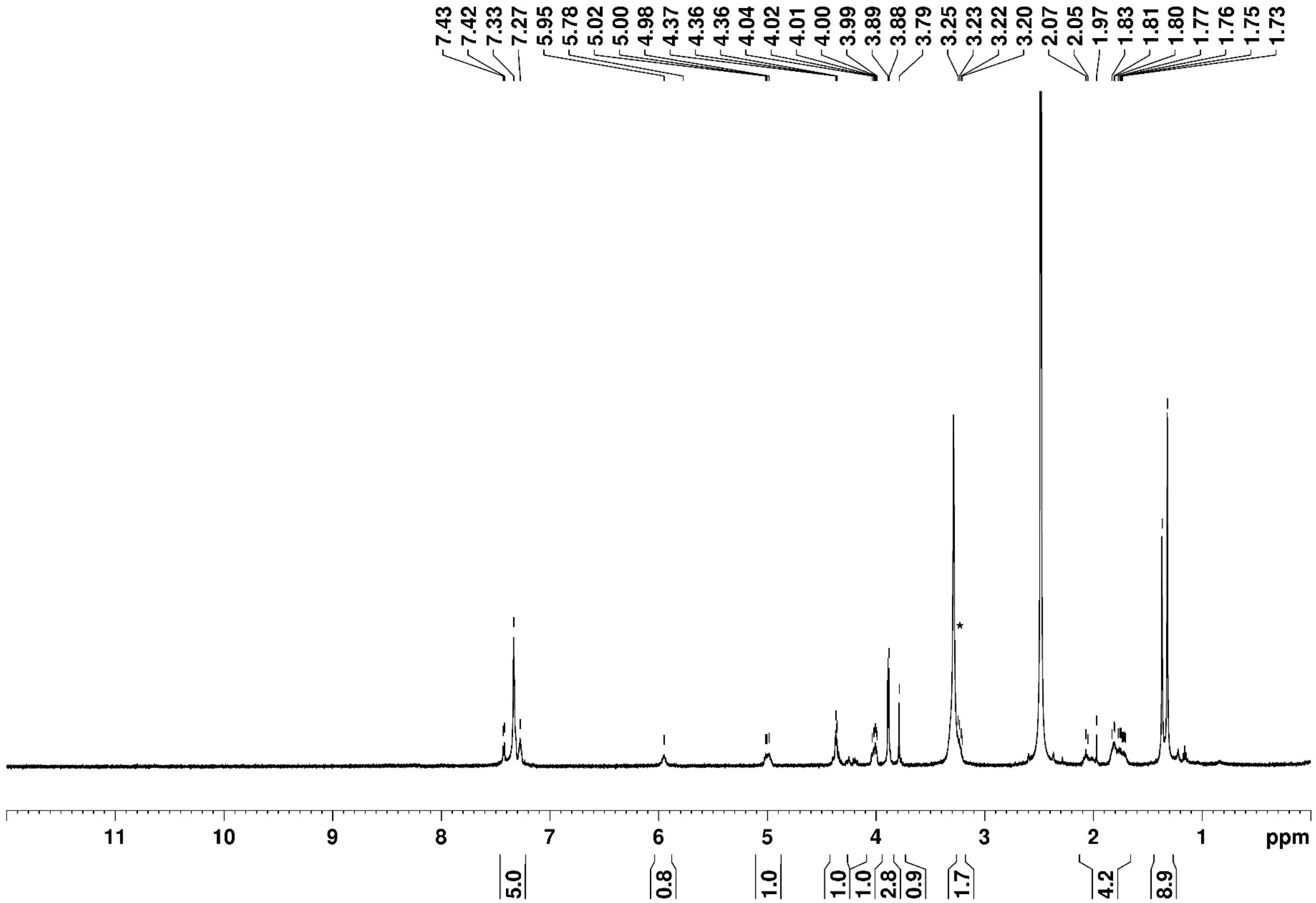

Figure S11. 2bP: ^{1}H NMR (400 MHz, DMSO-d$_{6}$, 298K)

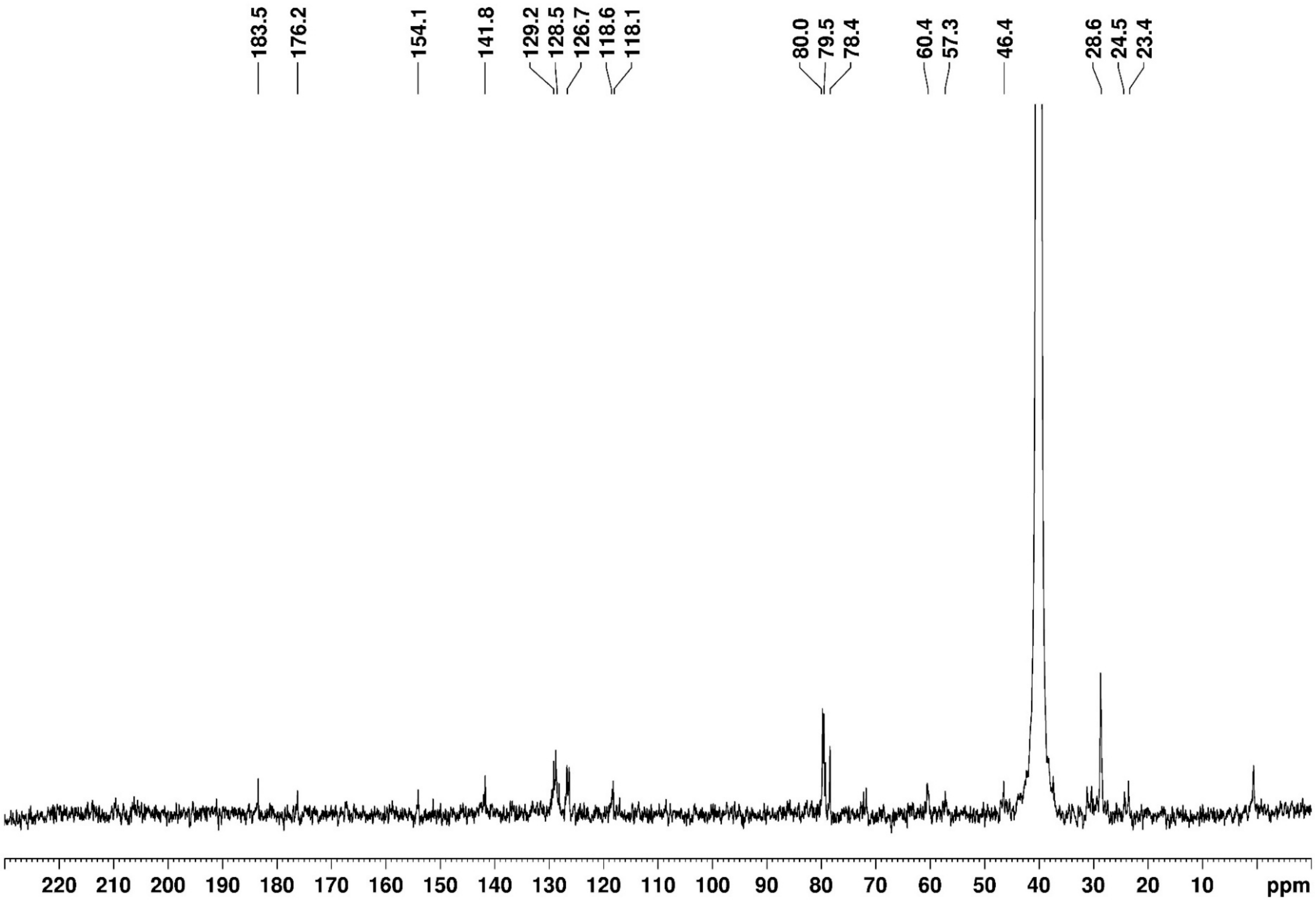

Figure S12. 2bP: ^{13}C NMR (100 MHz, DMSO-d_6, 298K)

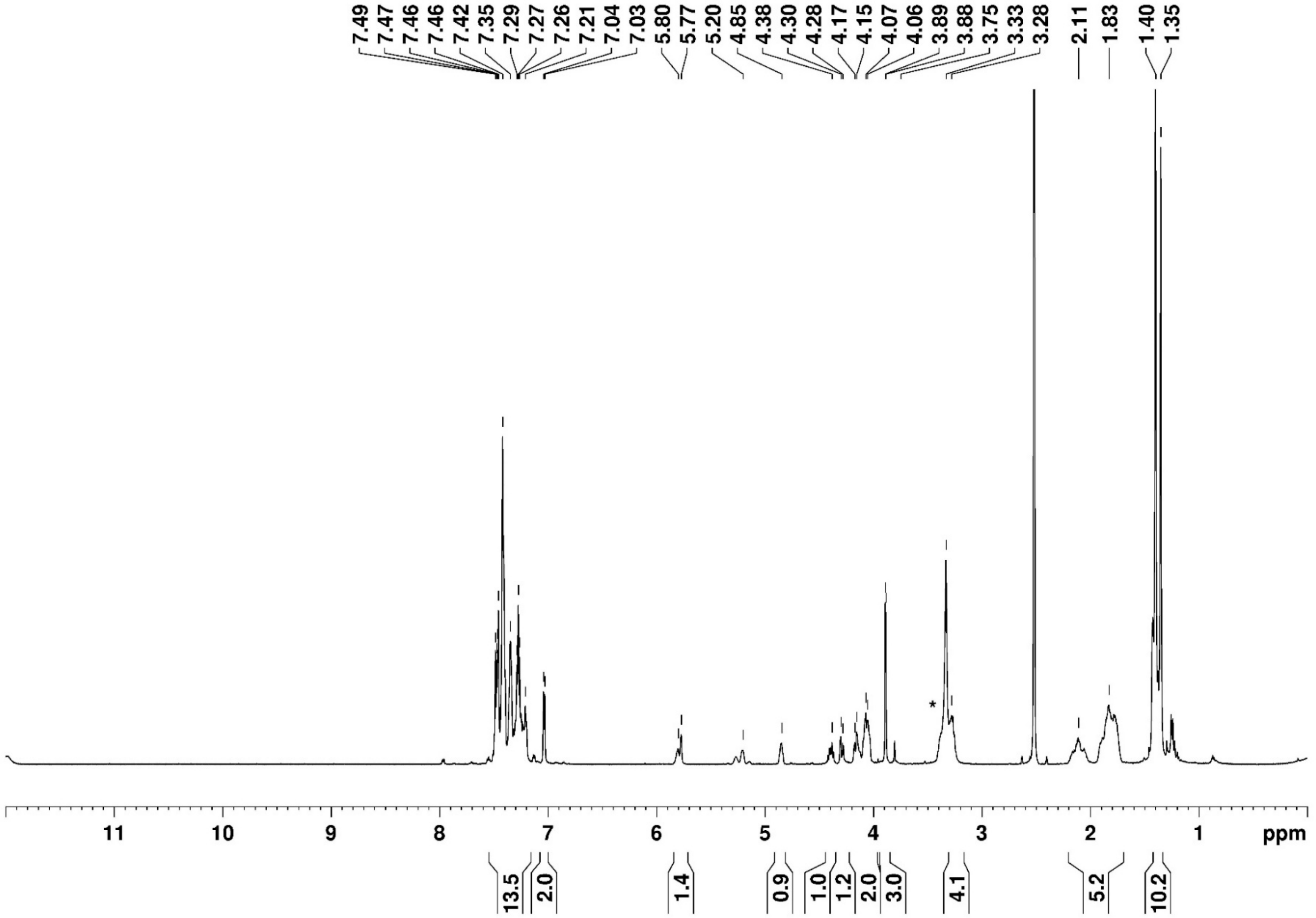

Figure S13. 3bP: ^{1}H NMR (400 MHz, DMSO-d$_6$, 298K)

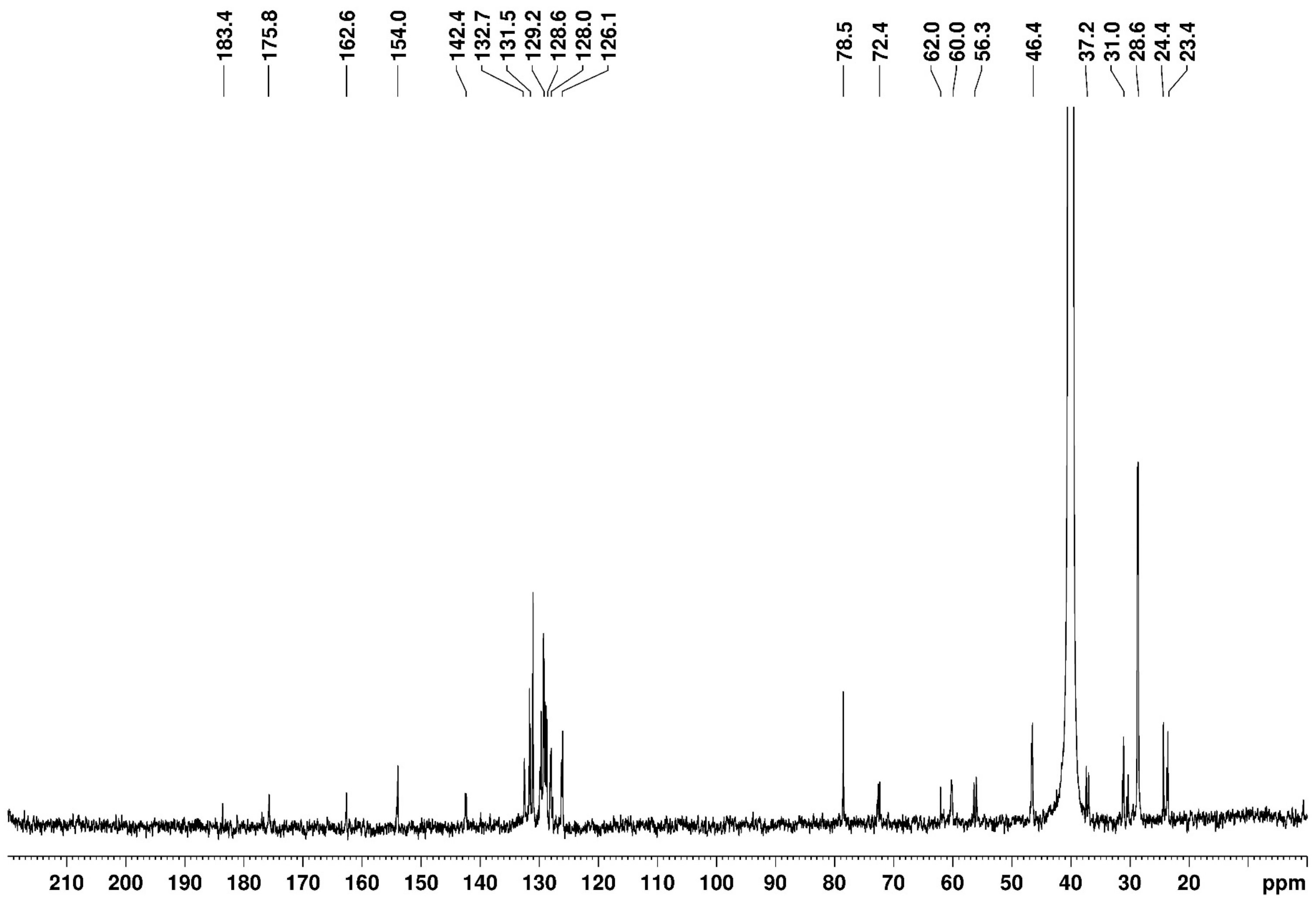

Figure S14. 3bP: ^{13}C NMR (100 MHz, DMSO-d_6, 298K)

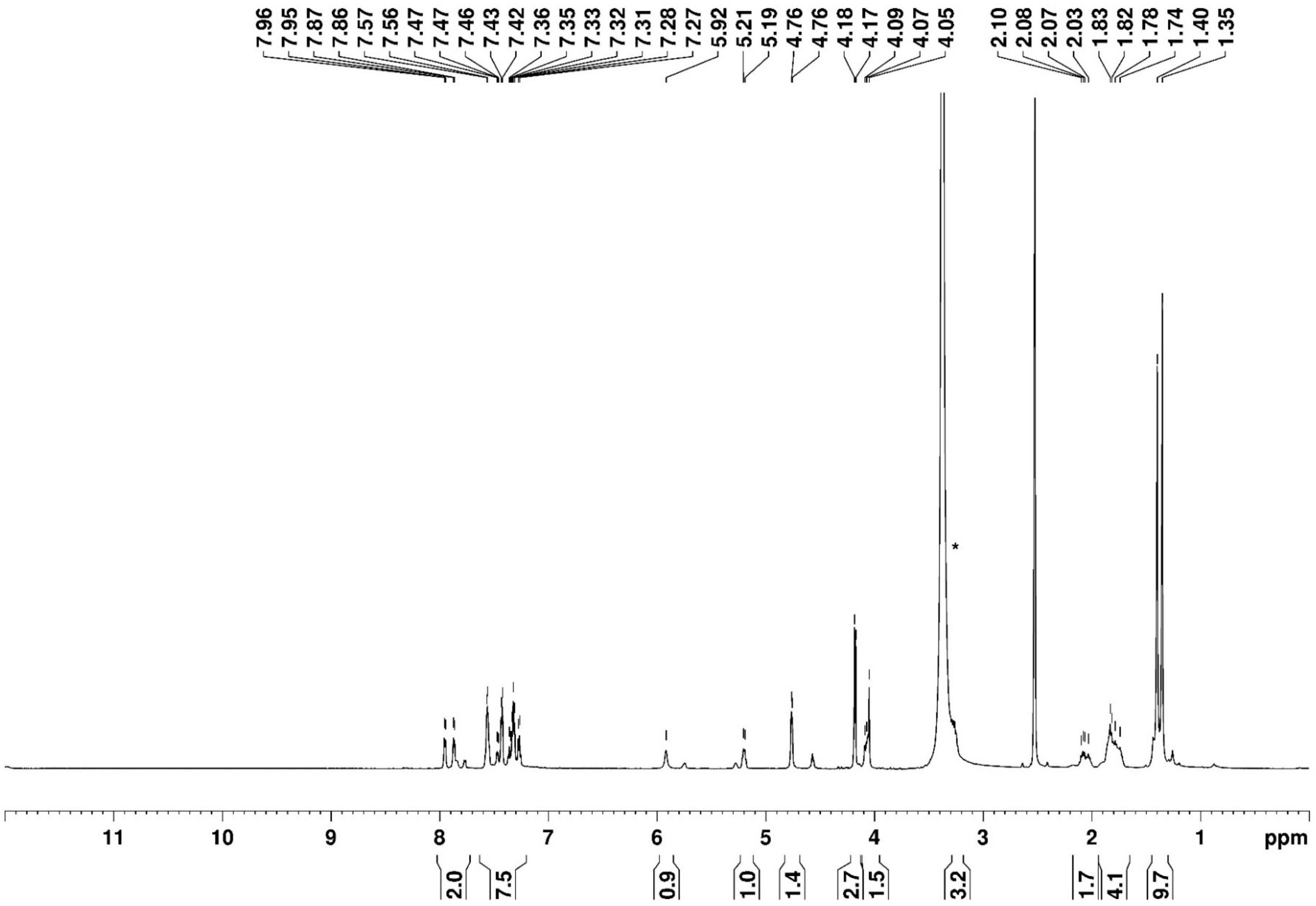

Figure S15. 4bP: ^{1}H NMR (400 MHz, DMSO-d$_6$, 298K)

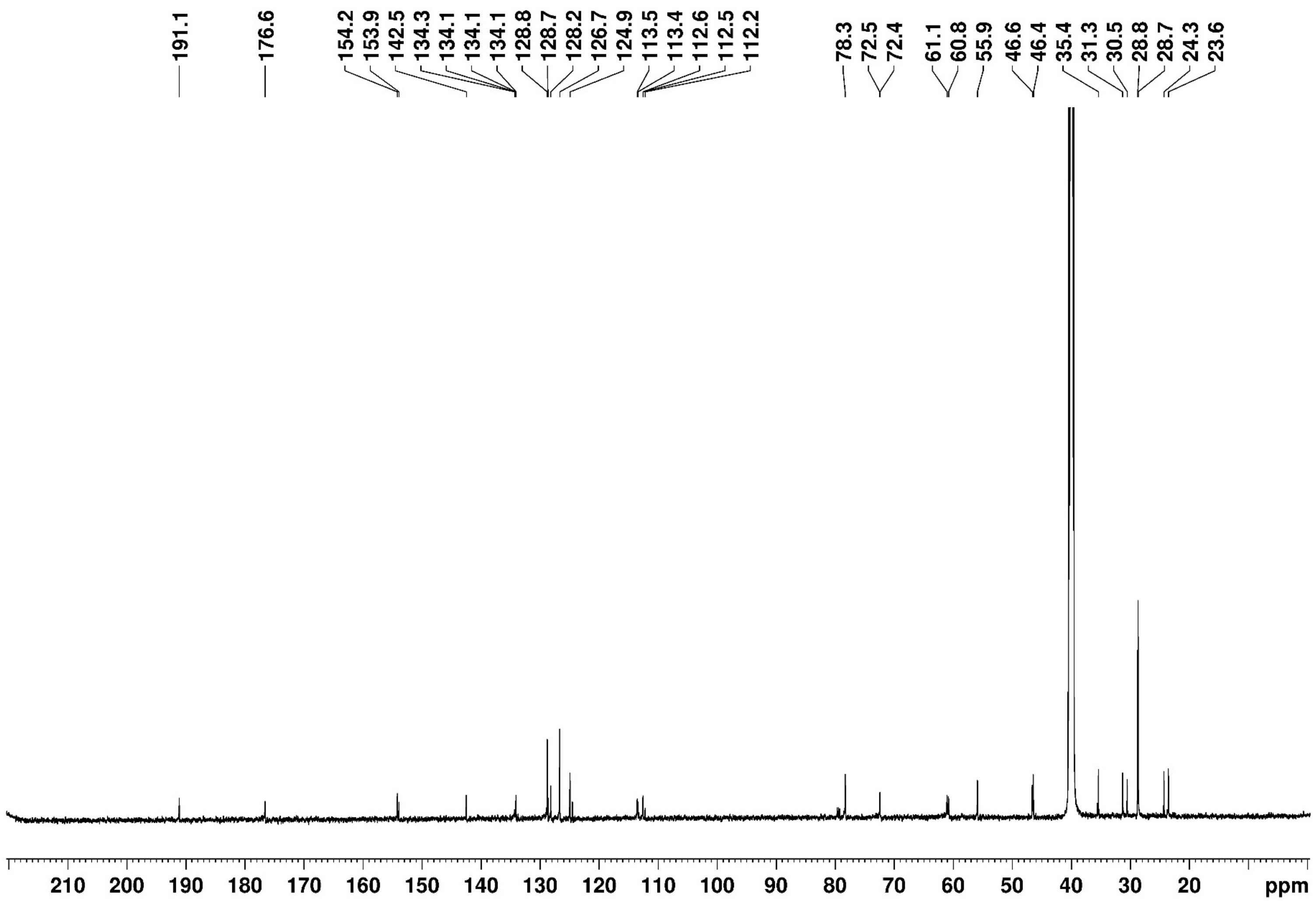

Figure S16. 4bP: ^{13}C NMR (100 MHz, DMSO-d_6, 298K)
